# The *Legionella* effector LtpM is a new type of phosphoinositide-activated glucosyltransferase

**DOI:** 10.1074/jbc.RA118.005952

**Published:** 2018-12-20

**Authors:** Nadezhda Levanova, Corinna Mattheis, Danielle Carson, Ka-Ning To, Thomas Jank, Gad Frankel, Klaus Aktories, Gunnar Neels Schroeder

**Affiliations:** From the ‡ Institute of Experimental and Clinical Pharmacology and Toxicology, Faculty of Medicine, University of Freiburg, D-79104 Freiburg, Germany,; the § MRC Centre for Molecular Bacteriology and Infection, Department of Life Sciences, Imperial College London, London SW7 2AZ, United Kingdom, and; the ¶ Wellcome-Wolfson Institute for Experimental Medicine, School of Medicine, Dentistry, and Biomedical Sciences, Queen's University Belfast, Belfast BT9 7BL, Northern Ireland, United Kingdom

**Keywords:** bacterial pathogenesis, infection, host-pathogen interaction, glycosyltransferase, protein secretion, effector, endosomal system, Legionella, phosphoinositide binding domain, type IV secretion system (T4SS)

## Abstract

*Legionella pneumophila* causes Legionnaires' disease, a severe form of pneumonia. *L. pneumophila* translocates more than 300 effectors into host cells via its Dot/Icm (Defective in organelle trafficking/Intracellular multiplication) type IV secretion system to enable its replication in target cells. Here, we studied the effector LtpM, which is encoded in a recombination hot spot in *L. pneumophila* Paris. We show that a C-terminal phosphoinositol 3-phosphate (PI3P)-binding domain, also found in otherwise unrelated effectors, targets LtpM to the *Legionella*-containing vacuole and to early and late endosomes. LtpM expression in yeast caused cytotoxicity. Sequence comparison and structural homology modeling of the N-terminal domain of LtpM uncovered a remote similarity to the glycosyltransferase (GT) toxin PaTox from the bacterium *Photorhabdus asymbiotica*; however, instead of the canonical DxD motif of GT-A type glycosyltransferases, essential for enzyme activity and divalent cation coordination, we found that a DxN motif is present in LtpM. Using UDP-glucose as sugar donor, we show that purified LtpM nevertheless exhibits glucohydrolase and autoglucosylation activity *in vitro* and demonstrate that PI3P binding activates LtpM's glucosyltransferase activity toward protein substrates. Substitution of the aspartate or the asparagine in the DxN motif abolished the activity of LtpM. Moreover, whereas all glycosyltransferase toxins and effectors identified so far depend on the presence of divalent cations, LtpM is active in their absence. Proteins containing LtpM-like GT domains are encoded in the genomes of other *L. pneumophila* isolates and species, suggesting that LtpM is the first member of a novel family of glycosyltransferase effectors employed to subvert hosts.

## Introduction

*Legionella pneumophila* and related species are Gram-negative bacteria, which colonize a wide variety of ecological niches, in particular aquatic environments (1). In these ecosystems, the constant interaction with protozoa, unicellular phagocytes that feed on bacteria, has driven the acquisition of virulence factors by *Legionella* spp., enabling them to evade predation and instead exploit the predators as replicative niches (2, 3). Respiratory infection occurs through accidental inhalation of bacteria-laden aerosols and can develop into a severe, potentially fatal pneumonia, called Legionnaires' disease (4). *Legionella* spp. account for 3–5% of hospitalized cases of pneumonia and a rising incidence of Legionnaires' disease in many countries, including the United States in recent years, highlighting *Legionella* spp. as an emerging threat for human health (5, 6).

In *L. pneumophila*, an essential virulence factor for the subversion of phagocytes and pathogenesis is the Defective in organelle trafficking/Intracellular multiplication (Dot/Icm)^5^ type IV secretion system (T4SS), which *L. pneumophila* uses to translocate more than 300 effector proteins into a host cell (7–9). The individual functions of most effectors remain unknown; but it has become clear that effectors modulate numerous processes, *e.g.* transcription, translation, ubiquitin-signaling, and vesicle trafficking (10, 11). Most importantly, the concerted action of effectors decouples the nascent *Legionella*-containing phagosome from the phago-lysosomal degradation pathway and remodels it into a unique organelle, the *Legionella*-containing vacuole (LCV) (12). The staggering number of Dot/Icm T4SS effectors is believed to provide the bacteria with the means to exploit a wide variety of diverse protozoan hosts (13). In addition, the discovery of multiple layers of manipulation of the small GTPase Rab1 through at least seven effectors (SidM, SidD, AnkX, Lem3, LepB, LidA, and PieE), resulting in tight spatio-temporal control of its activity, illustrates that the large effector arsenal also serves to fine-tune host cell processes (14, 15).

The effector repertoire expands by gene duplication giving rise to families of paralogue effectors, horizontal gene transfer between bacteria, and most likely, gene acquisition from eukaryotic hosts (16–19). Moreover, the detailed functional characterization of effectors, such as SidM (20–23), RavZ (24, 25), and SetA (26), revealed that many effectors are modular and consist of variable combinations of several functional domains. Deciphering the biochemical functions of these different effectors, including their numerous functional domains, is not only key to uncover how *Legionella* spp. subvert the host but also how the virulence factor reservoirs of these emerging human pathogens evolve.

Recombination hot spots in *L. pneumophila* genomes might play an important role in the biogenesis of new effectors (27). Among the proteins encoded in one of these hot spots is Lpp0356 (27), which we identified previously as putative Dot/Icm T4SS effector based on the presence of a C-terminal domain, which shares 64% identity with the phosphoinositide 3-phosphate (PI3P)-binding domain of the effector LtpD (Lpw_03701, WP_038837285.1) (28). Here, we characterized Lpp0356 in detail. We demonstrate that Lpp0356 is translocated by the Dot/Icm T4SS, and we therefore designate it *Legionella* translocated protein M (LtpM). We found that the N-terminal domain is a new type of glucosyltransferase, which does not rely on a catalytic DxD motif and metal ion co-factors like classical type A glycosyltransferases (GT-As), such as Lgt1 (29) and SetA (26). Moreover, we also found that the enzymatic activity is modulated by phospholipid binding through an interplay between the GT- and PI3P-binding domains.

## Results

### LtpM is a new Dot/Icm T4SS translocated effector, which localizes to the LCV

**Figure 1.**
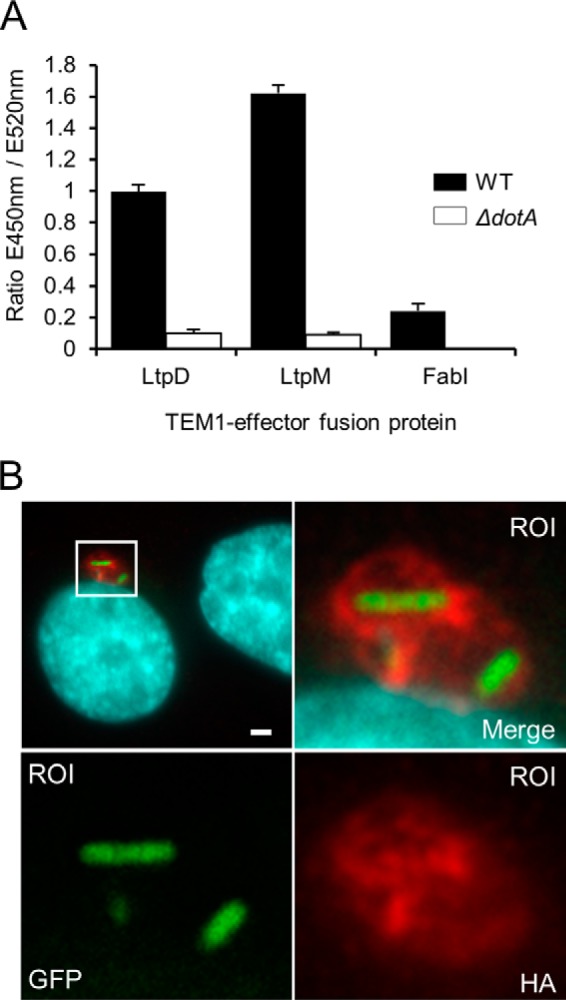
**LtpM is a Dot/Icm T4SS translocated effector, which localizes to the LCV.** *A,* β-lactamase (TEM1) translocation assays. *L. pneumophila* Paris WT (*black bars*) or T4S-deficient Δ*dotA* mutant (*white bars*) expressing TEM1-fused to LtpM were used to infect Raw264.7 macrophages (m.o.i. 40) for 1 h before addition of the cell-permeable CCF2-AM FRET TEM1 substrate. Bacteria expressing TEM1 fused to LtpD or the housekeeping protein FabI served as positive and negative controls, respectively. 3 h post-infection, fluorescence emissions of intact and TEM1-cleaved CCF2-AM substrate were recorded with a Fluostar Optima plate reader (410-nm (10-nm bandpass) excitation and 450- and 520-nm emission filters) and the emission (*E*) ratio as indicator for translocation rate calculated for each sample. *Error bars* represent standard deviations (S.D.). Results are representative of three independent experiments. *B,* IF micrographs of A549 lung epithelia cells infected with *L. pneumophila* Paris WT expressing GFP and LtpM-fused to four HA tags. 6 h post-infection, cells were fixed with PFA and then processed for IF microscopy using anti-HA antibody (*red*). DNA was visualized with DAPI (*cyan*). A region of interest (*ROI*) of the imaged cell was enlarged to show the LCV-like pattern of LtpM around the bacteria. Data are representative of at least three independent experiments. *Scale bar,* 2 μm.

To prove that LtpM is a Dot/Icm T4SS effector, we employed the β-lactamase (TEM1) translocation assay (30), which measures the change of fluorescence emission of a β-lactam Förster resonance energy transfer (FRET) reporter (CCF2-AM) upon cleavage by TEM1–effector fusion proteins that are delivered into the host cell cytoplasm. Infection of Raw264.7 macrophage–like cells with *L. pneumophila* Paris WT expressing TEM1–LtpM or -LtpD, but not the corresponding T4SS-deficient Δ*dotA* strains or WT bacteria expressing a TEM1 fusion to the housekeeping protein Fab1, resulted in a sharp increase of the ratio of the fluorescence signals of the cleaved β-lactam product over the substrate (Fig. 1*A*), showing that LtpM is translocated into host cells by the Dot/Icm T4SS.

To determine the localization of LtpM in infected host cells, we infected human lung epithelial A549 cells (Fig. 1*B*) or macrophage-like THP-1 or Raw264.7 cells (Fig. S1) with *L. pneumophila* Paris WT expressing LtpM fused to four hemagglutinin tags (4HA–LtpM), immunostained and analyzed the samples by immunofluorescence (IF) microscopy. 6 h post-infection, 4HA–LtpM was detected surrounding the WT bacteria in a pattern reminiscent of LCV-associated effectors such as SidM (15). These findings show that LtpM is translocated by the Dot/Icm T4SS and indicate that it localizes to the cytoplasmic leaflet of LCVs.

### LtpM is dispensable for replication of L. pneumophila in host cells

LtpM is found in *L. pneumophila* Paris and 48 other *L. pneumophila* strains (Table S1) and is highly conserved (>98% identity); however, it is not present in all sequenced strains, suggesting that it is not a core but an accessory effector and/or might be recently acquired. Notably, proteins showing partial homology to either the C-terminal putative PI3P-binding domain or the N-terminal domain of LtpM are found in hundreds of *L. pneumophila* isolates but also other *Legionella* spp. and few other γ-proteobacteria (Table S1), indicating that these domains might be common building blocks for effectors.

To determine the contribution of LtpM to intracellular survival and replication of *L. pneumophila* Paris, we generated a Δ*ltpM* deletion mutant and challenged THP-1 cells or the protozoan model *Dictyostelium discoideum* with WT or Δ*ltpM* mutant bacteria expressing fluorescent proteins. Continuous measurement of the increase in fluorescence revealed that *L. pneumophila* Δ*ltpM* was not attenuated in its ability to infect and replicate in the two hosts (Fig. S2), showing that LtpM is not essential for virulence.

### LtpM(469–639) binds PI3P with high affinity and targets LtpM to PI3P-containing membranes

**Figure 2.**
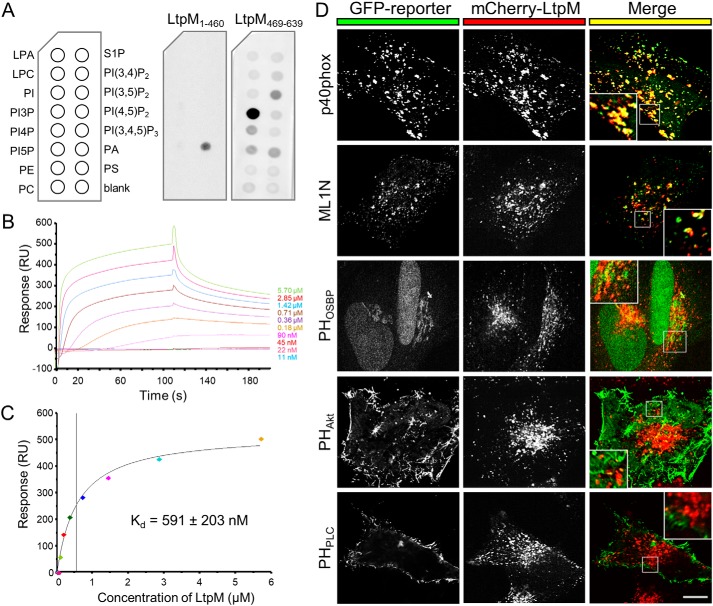
**C-terminal domain of LtpM specifically binds PI3P and targets LtpM to PI3P-containing membranes.** *A,* protein–phospholipid overlay assay with LtpM protein fragments, LtpM(1–460) and LtpM(469–639) (each 100 nm). Nitrocellulose membranes pre-spotted with 100 pmol of phospholipids were incubated with the indicated proteins and probed with an anti-LtpM serum. *Left lanes,* lysophosphatidic acid (*LPA*), lysophosphocholine (*LPC*), phosphatidylinositol (*PI*), phosphatidylinositol phosphate (*PIP*), phosphatidylethanolamine (*PE*), phosphatidylcholine (*PC*). *Right lanes,* sphingosine 1-phosphate (*S1P*), phosphatidylinositol phosphate (*PIP*), phosphatidic acid (*PA*), phosphatidylserine (*PS*). *B,* blank subtracted sensorgrams showing binding of LtpM to biotin-PI3P immobilized on an SA-Chip measured by surface plasmon resonance spectroscopy. LtpM was diluted in a 2-fold consecutive dilution series ranging from 5.7 μm to 11 nm and flowed over the chip surface for 110 s with subsequent washing steps of 500 s. *C,* equilibrium binding analysis indicates a *K_d_* = 591 ± 203 nm (S.D., *n* = 3). *B* and *C,* results are representative of three independent experiments. *D,* co-localization of LtpM with sensor domains for cellular phosphoinositides. LtpM co-localizes with PI3P and PI(3,5)P_2_-containing vesicles but not with the intracellular membranes containing PI4P, PI(4,5)P_2,_ PI(3,4)P_2_, or PI(3,4,5)P_3_. HeLa cells were transiently transfected with pmCherry–LtpM (*center, red*) and analyzed for co-localization with specific probes for cellular phosphoinositides (*left, green*, pEGFP): the p40^Phox^ PX domain of NADPH oxidase is specific for PI3P; the cytoplasmic domain ML1N of the receptor mucolipin-1 is specific for PI(3,5)P_2_; the PH domain of OSBP is specific for PI4P localizing at the Golgi/trans-Golgi network; the PH domain of AKT recognizes both PI(3,4)P_2_ and PI(3,4,5)P_3_; and the PH domain of PLCδ1 is specific for PI(4,5)P_2_. After 18 h, cells were fixed and analyzed with a confocal microscope. Co-localization is depicted in *yellow* (*right panel*). *Insets* show a magnification of the regions marked with *white lines. Scale bar,* 10 μm.

LtpM exhibits homology of its C terminus to the PI3P-binding domain of LtpD (28). To assess whether LtpM binds PIPs, we purified His_6_-tagged LtpM variants and performed protein–lipid overlay assays (26, 28, 31). This revealed strong binding of full-length LtpM and LtpM(469–639) to PI3P with high specificity, whereas LtpM(1–460) lacking the putative PI3P-binding domain showed no specific binding to any of the phosphoinositides (Fig. 2*A*; Fig. S3). To quantify the affinity of LtpM for PI3P, the biotin-conjugated lipid was immobilized on streptavidin-coated sensor chips, and the binding of purified LtpM was recorded by surface plasmon resonance spectroscopy. The analysis (Fig. 2*B*) showed dose-dependent binding of LtpM to the lipid anchor and allowed us to determine an equilibrium binding dissociation constant *K_d_* of 591 ± 203 nm (Fig. 2*C*), which is in the same range as the PI3P-binding affinity of the effector SetA (809 ± 51 nm (26)).

We next used a panel of GFP-tagged biosensors for different PIPs and confocal microscopy to analyze whether in transiently transfected HeLa cells LtpM was directed to membranes rich in a specific phosphoinositide phosphate (Fig. 2*D*). Ectopically-expressed mCherry–LtpM co-localized with GFP biosensors for PI3P (p40^Phox^ PX domain of NADPH oxidase) and PI(3,5)P_2_ (cytoplasmic domain ML1N of the receptor mucolipin 1) in vesicles throughout the cell, but not with sensors for PI4P (PH domain of oxysterol-binding protein 1 (OSBP)), PI(3,4)P_2_, and PI(3,4,5)P_3_ (PH domain of AKT) or PI(4,5)P_2_ (PH domain of PLCδ1). LtpM(1–462) showed diffuse cytoplasmic and nuclear localization, whereas LtpM(456–639) co-localized with a PI3P sensor as the full-length protein (Fig. S4). These data show that the last 170 amino acid residues of LtpM compose a high-affinity PI3P-binding domain, which is essential and sufficient to target LtpM to PI3P-containing membranes.

### LtpM shows remote similarity to glycosyltransferase toxins

**Figure 3.**
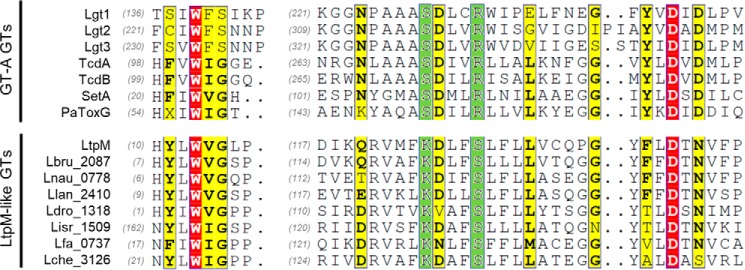
**LtpM shows sequence similarity to bacterial glycosyltransferase toxins.** Sequence alignment of bacterial GT-A GTs (Lgt1, Lgt2, and Lgt3 from *L. pneumophila*, TcdA and TcdB from *C. difficile*, SetA from *L. pneumophila*, and PaTox from *P. asymbiotica*) and the LtpM-like putative glycosyltransferases (LtpM-like GTs) from *Legionella* spp. (LtpM from *L. pneumophila*; Lbru_2087 from *L. brunensis*; Lnau_0778 from *L. nautarum*; Llan_2410 from *L. lansingensis*; Ldro_1318 from *L. drozanskii*; Lisr_1509 from *L. israelensis*; Lfa_0737 from *L. fallonii*; and Lche_3126 from *L. cherrii*). Amino acids strongly conserved among both groups are shown in *red*. Amino acids conserved only inside one of the group are shown in *green*. Variable amino acids are shown in *yellow*.

Next, we performed a bioinformatics analysis of LtpM(1–460) to uncover conserved sequence motifs, revealing the presence of three putative ankyrin repeats preceding the PI3P-binding domain (LtpM(329–435)). Ankyrin repeats are typically eukaryotic domains involved in protein–protein interactions, but are also frequently found in *Legionella* effectors (32). Moreover, sequence and structural homology searches using NCBI BlastP and Phyre^2^ indicated a remote similarity of LtpM(9–145) to the glycosyltransferase (GT) domain of the insecticidal protein toxin PaTox of the entomopathogen *Photorhabdus asymbiotica* (33) and to the *Legionella* glycosyltransferase effector SetA (26, 34). Alignment of LtpM with the GT domains of selected well-characterized GTs and modeling of LtpM(9–145) on the crystal structure of PaTox-G from *P. asymbiotica* (Fig. 3, Fig. S5, *A* and *B*, File S1) (33) revealed that conserved amino acid residues of LtpM are positioned around the catalytic pocket of PaTox. Among the conserved residues are aspartic acid 124 and tryptophan 13, which correspond to residues of PaTox implicated in stabilizing the interaction with the UDP-sugar donor. Remarkably, a characteristic DxD motif, which plays a critical role in catalysis in PaTox (33) and other members of the GT-A family of glycosyltransferases (35), is not conserved in LtpM. This DxD motif interacts with the hydroxyl groups of the UDP-sugar and coordinates an essential divalent metal ion cofactor, *e.g.* Mg^2+^ or Mn^2+^. In LtpM, the second aspartate is replaced by an asparagine (Asn-142).

### LtpM has glucohydrolase and auto-glucosyltransferase activity

**Figure 4.**
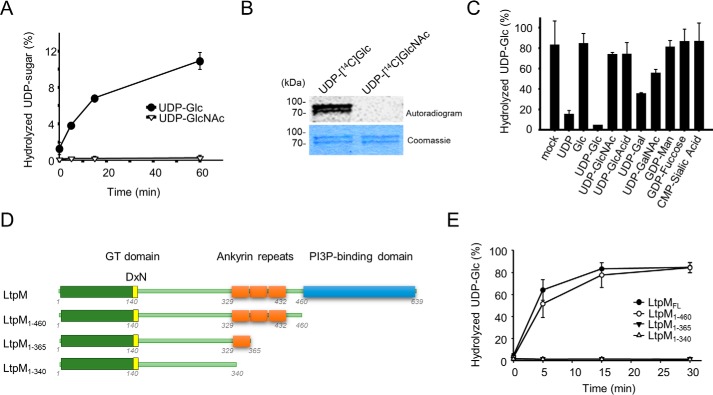
**LtpM specifically hydrolyzes UDP-Glc *in vitro* and autoglucosylates.** *A,* time course of a UDP-sugar hydrolase activity using LtpM (1 μm) in a buffer containing 50 mm Hepes (pH 7.5), 150 mm KCl, 1 mm MnCl_2_, 2 mm MgCl_2_ at 30 °C. The reaction components were separated by PEI-TLC, and products were detected by autoradiography. *B,* autoglucosylation of LtpM. LtpM glucosylates itself with UDP-[^14^C]Glc but not with UDP-[^14^C]GlcNAc. Reaction was performed as in *A*, and products were separated in SDS-PAGE and detected by autoradiography. An autoradiogram (*top panel*) and a Coomassie-stained SDS-PAGE (*bottom panel*) are shown. *C,* determination of the donor substrate specificity. Hydrolysis of UDP-[^14^C]Glc by LtpM in presence of 100-fold excess of cold nucleotide sugars. UDP-Glc and UDP inhibit hydrolysis of UDP-[^14^C]Glc. *B* and *C, error bars* represent S.D. of three technical replicates. Results are representative of three independent experiments. *D,* schematic representation of the truncated LtpM variants used in this study. *E,* time course of UDP-glucose hydrolase activity of LtpM truncations. Hydrolysis of UDP-[^14^C]glucose (10 μm) was performed with LtpM_FL_, LtpM(1–460), LtpM(1–365), or LtpM(1–340) (each 1 μm) at 30 °C in a buffer containing 50 mm Hepes (pH 7.5), 10 mm DTT, 2 mm MgCl_2_ and 1 mm MnCl_2_. At the indicated time points, the products were separated by TLC and analyzed autoradiographically. *Error bars* represent standard deviations of three independent experiments.

The results of the bioinformatics analysis prompted us to determine whether LtpM possesses glucohydrolase and/or glucosyltransferase activity. All glycosyltransferase toxins identified so far, including PaTox and the previously characterized *Legionella* GT effectors SetA and Lgt1–3, use the nucleotide sugar donors UDP-GlcNAc (UDP-GlcNAc) or UDP-glucose (UDP-Glc) to glycosylate protein targets (36). In the absence of protein targets most GTs hydrolyze UDP-sugar donors (35, 37). To test whether LtpM displays glycohydrolase activity, recombinant His_6_-tagged LtpM was incubated with radiolabeled UDP-^14^C-sugars, and the release of ^14^C-sugars was measured by autoradiography of thin layer chromatograms (TLCs) of the reaction mixtures. These experiments revealed that LtpM causes hydrolysis of UDP-[^14^C]glucose but not of UDP-[^14^C]GlcNAc (Fig. 4*A*).

Hydrolysis of UDP-[^14^C]glucose by SetA was previously found to be accompanied by auto-glucosylation (26). Also, LtpM catalyzed an auto-glucosylation reaction in the presence of UDP-[^14^C]glucose but not with UDP-[^14^C]GlcNAc (Fig. 4*B*).

To further assess the sugar donor specificity of LtpM, the ability of a panel of unlabeled nucleotide sugars to outcompete UDP-[^14^C]glucose was analyzed (Fig. 4*C*). Addition of a 100-fold excess of UDP-glucose nearly completely abolished the release of [^14^C]glucose by LtpM. Also, UDP caused an inhibition of the reaction, whereas other sugar donors exerted no effect or only moderate effects on hydrolysis, suggesting that UDP-glucose is the preferred sugar substrate for LtpM.

We next investigated the role of the PI3P-binding domain and the ankyrin repeats for the glucohydrolase activity of LtpM (Fig. 4, *D* and *E*). This showed that LtpM(1–460), lacking the PI3P-binding domain, retained full activity. Shorter LtpM fragments were expressed but showed some signs of instability (Fig. S5*C*) and completely lost the glucohydrolase activity, suggesting that the ankyrin repeat region is important for the function and stability of the enzyme.

### PI3P increases the glucosyltransferase activity of LtpM

**Figure 5.**
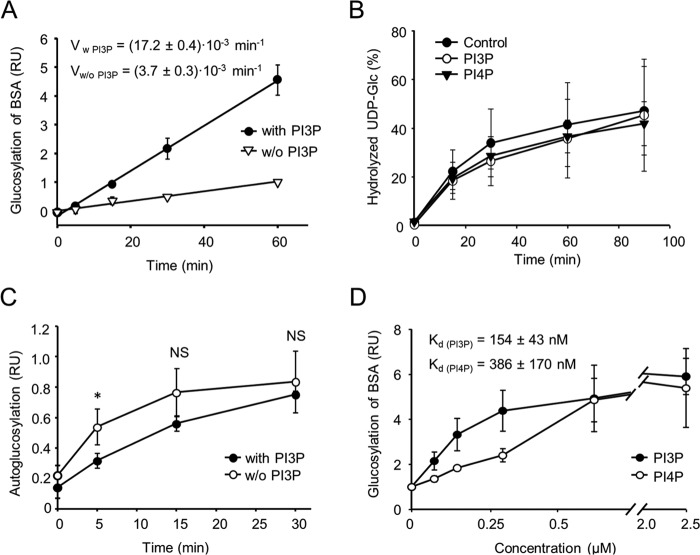
**PI3P activates the glucosyltransferase activity of LtpM.** *A,* time course of glucosylation of BSA with or without 1 μm PI3P. BSA was used as artificial glycosyl-acceptor substrate of LtpM. BSA was incubated with LtpM (25 nm) in presence of 10 μm UDP-[^14^C]glucose with or without 1 μm PI3P in a buffer containing 50 mm Hepes, 1 mm MnCl_2_, 2 mm MgCl_2_ at 30 °C for the indicated time. The modified proteins were separated by SDS-PAGE, dried, and detected radiographically. Individual measurements were normalized to the value recorded for the BSA glucosylation after a 60-min reaction without PI3P. *B,* UDP-glucohydrolase activity of LtpM is independent of PI3P binding. Time course of UDP-[^14^C]Glc hydrolysis by LtpM in presence of 10 μm PI3P (*open circles*) or PI4P (*black triangles*) or without any PIPs (*control, black circles*). UDP-[^14^C]Glc (10 μm) was incubated with LtpM (200 nm) in a buffer containing 50 mm Hepes, 1 mm MnCl_2_, 2 mm MgCl_2_, and 10 mm DTT at 30 °C. The reaction components were separated by TLC, and products were detected by autoradiography. *C,* autoglucosylation of LtpM is inhibited by PI3P. Time course of LtpM autoglucosylation with or without 10 μm PI3P. LtpM (each 4 μm) was incubated with UDP-[^14^C]glucose (10 μm) in a buffer containing 50 mm Hepes, 1 mm MnCl_2_, 2 mm MgCl_2_, and 10 mm DTT at 30 °C. Individual measurements were normalized to the value recorded for LtpM autoglucosylation after a 60-min reaction with PI3P. *NS,* not significant; *, *p* ≤ 0.05. *D,* activation of GT activity of LtpM in the presence of increasing concentrations of PI3P or PI4P. Reactions were performed as in *B*. Individual measurements were normalized to the value recorded for the BSA glucosylation without any PIP added. *A–D,* data represent the mean of three independent experiments. *Error bars* represent standard deviations. *RU,* relative units; *V*, velocity.

We next asked whether LtpM can glucosylate artificial substrates and whether this is influenced by phosphoinositide binding. LtpM modified the artificial substrate BSA, and the rate of the glucosylation was increased up to 5-fold in the presence of PI3P (Fig. 5*A*). At the same time, the glucohydrolase activity was not affected by addition of PI3P or PI4P (Fig. 5*B*). The autoglucosylation of the enzyme was decreased by the addition of PI3P (Fig. 5*C*). The concentration–effect curves of influence of PI3P and PI4P on the glucosyltransferase activity of LtpM with BSA as substrate revealed a higher potency of PI3P as compared with PI4P. At high concentrations, PI4P reached the same level of activation as PI3P (Fig. 5*D*). These data show that phosphoinositide binding enhances the transfer of the sugar-moiety by LtpM onto target proteins.

### LtpM possesses glucohydrolase and glucosyltransferase activity in the absence of cations

**Figure 6.**
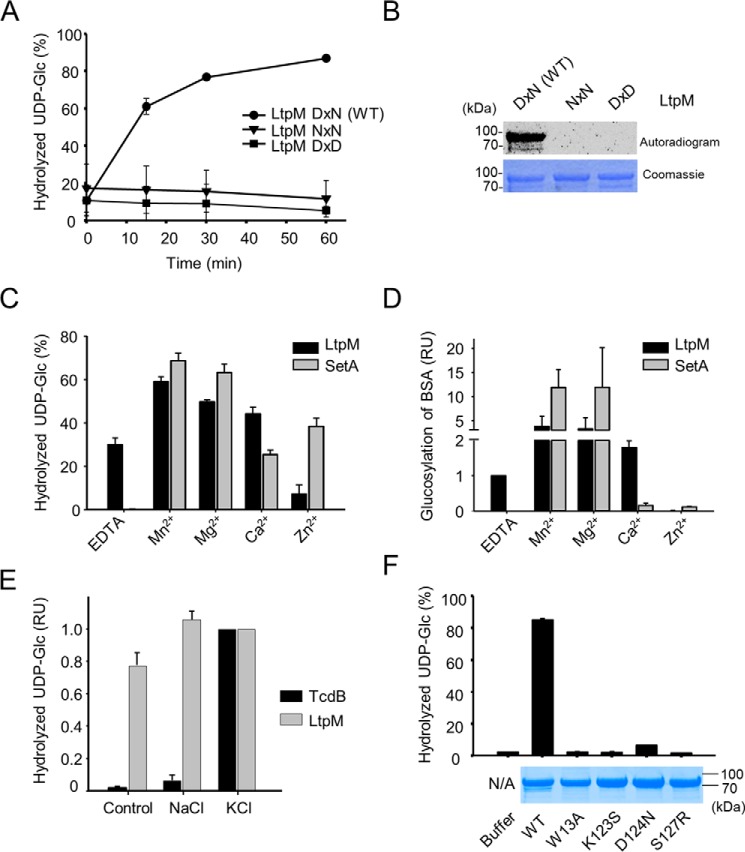
**LtpM is a metal ion-independent glucosyltransferase.** *A,* LtpM WT, but not the DxD and NxN mutants, hydrolyzes UDP-glucose *in vitro*. Time course of UDP-glucose hydrolysis using LtpM, LtpM NxN or LtpM, DxD mutants (1 μm each) is shown. Reactions were performed for the indicated time at 30 °C in a buffer containing 10 μm UDP-[^14^C]Glc, 50 mm Hepes (pH 7.5), 10 mm DTT, 2 mm MgCl_2_, and 1 mm MnCl_2_. The reaction components were separated by TLC, and products were detected by autoradiography. *B,* autoglucosylation of LtpM. The WT enzyme, but not NxN and DxD mutants of LtpM, glucosylates itself with UDP-[^14^C]Glc. Reactions were performed as in *A*, and products were separated in SDS-PAGE and detected by autoradiography. An autoradiogram (*top panel*) and a Coomassie-stained gel (*bottom panel*) are shown. *C,* UDP-glucose hydrolase activity of LtpM or SetA (1 μm each) in the presence of EDTA (100 μm) with or without addition of an excess (1 mm) of MnCl_2_, MgCl_2_, CaCl_2_, or ZnCl_2_. Reactions were performed at 30 °C for 30 min in a buffer containing 10 μm UDP-[^14^C]Glc, 50 mm Hepes (pH 7.5), 10 mm DTT. *D,* glucosylation of BSA (2.5 μg) by LtpM or SetA (25 nm each) in the presence of EDTA (1 mm) with or without addition of an excess (3 mm) of the indicated divalent ions (MnCl_2_, MgCl_2_, CaCl_2_, or ZnCl_2_). Reactions were performed at 30 °C for 40 min in a buffer containing 1 μm PI3P, 10 μm UDP-[^14^C]Glc, 50 mm Hepes (pH 7.5), 10 mm DTT. *E,* hydrolysis of UDP-glucose by LtpM and GT domain of TcdB in the presence of Na^+^ or K^+^. TcdB, but not LtpM, needs K^+^ and Mg^2+^ for activation. Reactions were performed at 30 °C in a buffer containing 50 mm Hepes (pH 7.5), 1 mm EDTA, 10 mm DTT, 3 mm MgCl_2_, 100 mm NaCl, or KCl. Individual measurements were normalized to the value recorded for the reaction in the presence of 100 mm KCl. *F, top,* hydrolysis of UDP-glucose by WT His-tagged LtpM (*WT*) or its mutant variants W13A, K123S, D124N, or S127R. Reactions were performed as in *A* for 30 min. *Bottom,* Coomassie-stained gel loaded with 5 μg of the corresponding proteins used in the reactions. *A, B,* and *F, error bars* indicate standard deviations of two technical replicates. Data are representative of three independent experiments. *C–E, error bars* indicate standard deviations of three independent experiments.

The hallmark of type A GTs is the presence of the DxD motif, which coordinates a divalent metal cation and UDP-glucose. The degeneration of the DxD to a DxN motif in LtpM (Fig. 3) is unique as compared with all other glycosyltransferase toxins. To clarify the role of the DxN motif of LtpM, we generated the D140N mutant, resulting in an NxN motif, which is known to inhibit the enzyme activity of many toxin glycosyltransferases (36). LtpM NxN exhibited no glucohydrolase and glucotransferase activity. Furthermore, we changed asparagine 142 to aspartate to restore the classical DxD motif. Also, this substitution rendered the enzyme inactive in glucohydrolase and auto-glucosylation autoradiography assays (Fig. 6, *A* and *B*), indicating that each residue of the DxN motif is critical for the function of the active site of LtpM.

The lack of the classical DxD motif does not exclude that LtpM still relies on a divalent metal ion cofactor for catalysis. To probe this, glucohydrolase and glucosyltransferase assays were carried out in the absence of added cations, in the presence of the chelator EDTA, or with added Mg^2+^, Mn^2+^, Ca^2+^, or Zn^2+^. As a control, we used SetA, which strictly depends on Mn^2+^ or Mg^2+^. In the presence of 100 μm EDTA, the glucohydrolase activity of SetA was completely inhibited, whereas LtpM exhibited robust activity (Fig. 6*C*). Addition of increasing amounts of MnCl_2_, MgCl_2_, CaCl_2_, or ZnCl_2_ up to equimolar concentration with EDTA had a minimal enhancing effect on the activity of LtpM (Fig. S6, *A–D*). Over the same concentration range, the activity of SetA strongly increased to match and even surpass LtpM in the presence of equimolar MnCl_2_ or MgCl_2_ (Fig. S6, *A–D*). A further increase in the concentrations of any of the four metal ions further enhanced the glucohydrolase activity of SetA. Excess of MgCl_2_, MnCl_2_, and CaCl_2_ also led to a moderate increase of the activity LtpM; but further addition of ZnCl_2_ reduced its activity (Fig. 6*C* and Fig. S6, *A–D*).

We next studied the metal dependence of the glucosyltransferase activity with the artificial substrate BSA (Fig. 6*D*; Fig. S7). The glucosyltransferase activity of SetA was completely dependent on divalent cations and only observed when at least equimolar or excess amounts of the metal ions over EDTA were present (Fig. S7). In contrast, LtpM exhibited glucosyltransferase activity in the presence of 1 mm EDTA without added divalent cations. This robust glucosylation of BSA by LtpM was further increased when the concentrations of MgCl_2_, MnCl_2_, and CaCl_2_ exceeded that of EDTA but was inhibited by ZnCl_2_.

We also tested the effects of the monovalent cations K^+^ and Na^+^. The activity of the GT domain of *Clostridium difficile* toxin B (TcdB) is known to strongly increase by addition of K^+^. LtpM was not affected by the presence or absence of monovalent ions (Fig. 6*E*). Taken together, LtpM differs from SetA in its divalent cation dependence and represents a new type of GT that possesses enzyme activity in the absence of divalent metal ions.

To gain further insights into the catalytic mechanism of LtpM, we mutated conserved amino acid residues, which were implicated in sugar-donor binding and catalysis in other type A GTs, and we determined the glucohydrolase activity of the mutants. Change of tryptophan 13 to alanine or aspartate 124 into asparagine resulted in complete loss of enzyme activity (Fig. 6*F*), demonstrating their catalytic or structural importance. This loss of activity was not due to the loss of protein stability as demonstrated by SDS-PAGE analysis. Moreover, various highly conserved amino acids found in classical toxin glycosyltransferases are changed in LtpM and related glycosyltransferases (Fig. 3), *e.g.* lysine 123 and serine 127 of LtpM substitute otherwise conserved serine and arginine residues. We generated LtpM K123S and S127R mutants and analyzed their glycohydrolase activity. Both mutants were unable to hydrolyze UDP-glucose indicating a crucial role of lysine 123 and serine 127 in the catalytic activity of LtpM. Taken together, the data show that the active site of LtpM is made up of a new combination of amino acid residues, enabling metal-independent glucosyltransferase activity.

### Ectopic expression of LtpM impacts viability of eukaryotic cells

**Figure 7.**
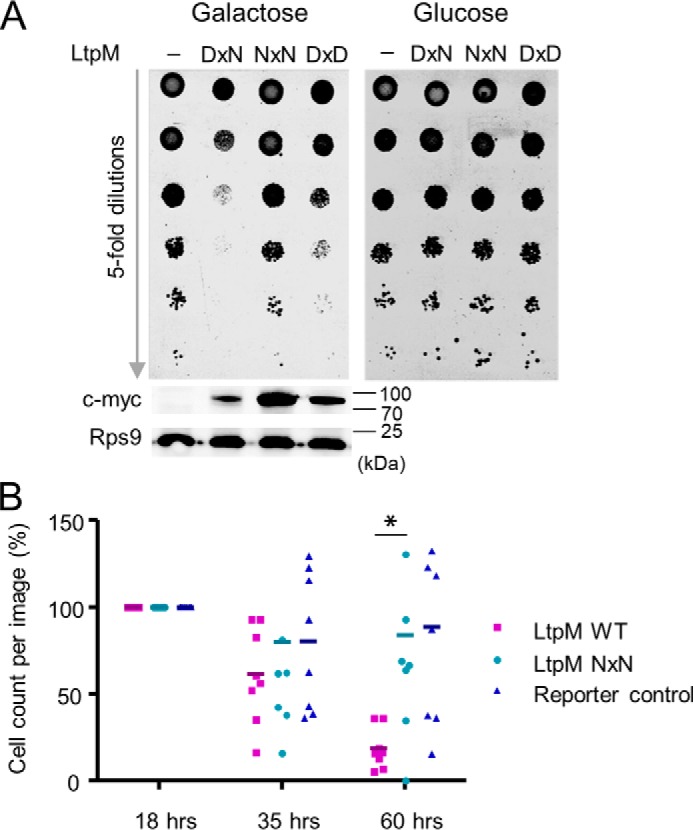
**Toxicity in mammalian cells and yeast.** *A,* spot-test assay of growth phenotypes of *S. cerevisiae*. LtpM inhibits growth of yeast. Exchange of the DxN motif to NxN leads to the loss of toxicity. A DxD mutant demonstrates lower toxicity compared with the WT. Yeast were transformed with pESC-His (−) or pESC-His-based plasmids coding for WT LtpM (DxN) and LtpM mutants: D140N (NxN) and N142D. Western blotting demonstrates production of c-Myc–tagged LtpM and LtpM mutants induced by galactose in *S. cerevisiae* strains. Antibodies against a ribosomal protein Rps9 were used as an input control of the lysates. *B,* LtpM represses growth of HeLa cells. The number of attached HeLa cells expressing LtpM WT (*magenta*) is reduced compared with the cells expressing LtpM NxN (*cyan*) or fluorescence reporter alone (*blue*). *Graph* showing count of attached transfected cells per image over the indicated time. HeLa cells were transiently transfected with pmCherry–LtpM, pmCherry–LtpM NxN, or pmCherry (reporter control). Time-lapse movies of the transfected cells were started 18 h post-transfection and were recorded for another 42 h using similar fields of view. The number of attached transfected cells per image were counted, and values were normalized to the values referred to the time point 18 h (start of the observation). Values for the individual data points, the mean values, and standard deviation (*S.D.*) of *n* = 8 biological replicates are shown. Data are representative of three independent experiments. *, *p* ≤ 0.05.

After establishing the GT activity of LtpM, we set out to elucidate its function. As we did not observe any impact of the chromosomal deletion of *ltpM* on intracellular growth of *L. pneumophila* (Fig. S2), we focused our effort on LtpM expression assays. The *Legionella* GT effectors Lgt1 and SetA were shown to be cytotoxic when expressed or delivered into eukaryotic cells (26, 38). To study the cytotoxicity of LtpM, we used an inducible system to express LtpM, LtpM NxN, or LtpM DxD mutants in the budding yeast *Saccharomyces cerevisiae*. Plating of serial dilutions of the yeast showed that induction of WT LtpM but not of the LtpM NxN mutant inhibited growth (Fig. 7*A*). The DxD mutant showed a very slight inhibitory effect on growth. The effects were not due to reduced expression of the LtpM mutants as Western blottings revealed equal or even higher protein levels. Consistent with the glucohydrolase assays (Fig. 4*E*), expression of LtpM fragments in the yeast (Fig. S8) demonstrated that LtpM(1–460) was the minimal domain required for the growth inhibition.

Next, we assessed the effect of ectopic expression of mCherry-tagged LtpM or inactive LtpM-NxN on cell viability using time-lapse microscopy. This did not reveal any immediate cytotoxicity or differences between the two LtpM variants within the first 2 days post-transfection of HeLa cells; however, 60 h post-transfection significantly (*p* = 0.026) less viable cells were observed in the pmCherry–LtpM-transfected sample compared with the pmCherry–LtpM NxN or pmCherry-transfected controls (Fig. 7*B*). The data show that LtpM uses its GT domain to target cellular processes, which are important for cell viability/growth in yeast and mammalian cells.

### LtpM blocks the microtubule-dependent movement of endosomes

**Figure 8.**
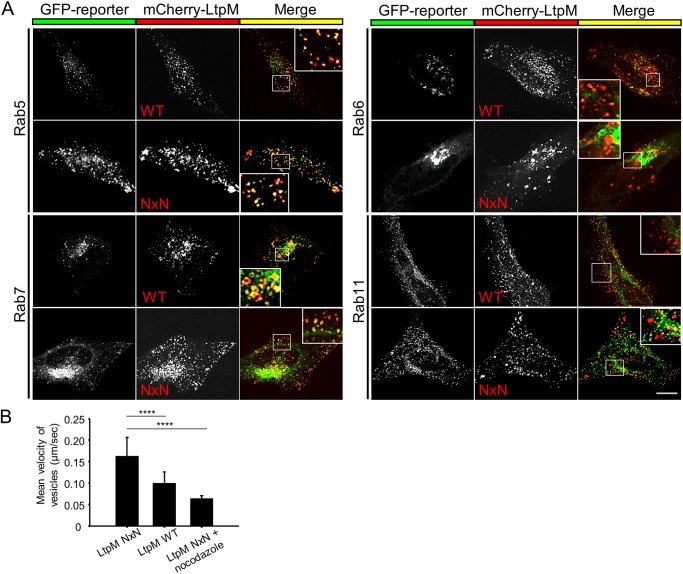
**LtpM modulates endosomal vesicle trafficking in HeLa cells.** *A,* HeLa cells were transiently transfected with pmCherry–LtpM or pmCherry–LtpM NxN (*center, red*) and analyzed for co-localization with specific marker proteins for vesicular compartments (*left, green*): pEGFP-Rab5; pEGFP-Rab7A; pEGFP-Rab6; and pEGFP-Rab11. After 18 h, cells were fixed and analyzed with a confocal microscope. Images are representative of at least three independent experiments. Co-localization is depicted in *yellow* (*right panel*). *Insets* show a magnification of the regions marked with *white lines. Scale bar,* 10 μm. *B,* velocity of LtpM-labeled vesicles was analyzed in HeLa cells. HeLa cells were transfected with pmCherry–LtpM WT or -LtpM NxN for 18 h. Nocodazole-treated cells were used as a control. Nocodazole (10 μm) was applied for 2 h before microscopy. Single cell microscopy time-lapse movies (3 min, 1 frame/s) were recorded, and the velocity of LtpM-labeled vesicles was analyzed (ImageJ Software). The mean velocity of the vesicles labeled with WT LtpM is significantly lower than the mean velocity of the vesicles labeled with the enzymatically inactive NxN mutant. For the quantification of the experiment, at least 1000 tracks per cell were analyzed. *Error bars* indicate standard deviations of at least seven biological replicates. ****, *p* ≤ 0.0001.

Lgt1 causes toxicity by inhibiting translation; however, LtpM did not show a similar effect in *in vitro* translation assays (Fig. S9). As LtpM localizes to PI3P-containing vesicles, we hypothesized that it might exert its effect on the cell by modulating targets in these compartments (Fig. 2*D*). To delineate the nature of the LtpM-containing vesicles, we performed co-transfections of pmCherry–LtpM with plasmids encoding GFP-tagged Rab GTPases as markers of different membranes. Confocal immunofluorescence microscopy showed that mCherry–LtpM and -LtpM NxN co-localized with the early and late endosomal GTPases Rab5 and Rab7 but not with Rab6 and Rab11 (Fig. 8*A*), which characterize recycling endosomes that mediate recycling of endocytosed cargo back to the cell surface.

We next determined whether LtpM interfered with different membrane trafficking pathways, in which early and late endosomes participate. Ectopic expression of mCherry–LtpM neither affected the retrograde transport of cholera toxin from the cell surface via endosomes to the Golgi apparatus (Fig. S10, *A* and *B*) nor caused a significant defect in the recycling of the iron carrier transferrin from endosomes to the cell surface (Fig. S10*C*).

Furthermore, we visualized the movement of the vesicles loaded with mCherry–LtpM or -LtpM NxN in HeLa cells. We recorded time-lapse movies of the living cells and analyzed tracks of the vesicles (Fig. S11*A* and Movies S1–S4). LtpM-labeled vesicles moved with the mean velocity of 0.10 ± 0.03 μm/s (*n* = 9) for the WT LtpM and 0.16 ± 0.04 μm/s (*n* = 15) for the inactive mutant showing that significantly less vesicle movement is present in LtpM-expressing cells compared with the NxN mutant (Fig. 8*B*). Co-expression of mCherry–LtpM and enhanced GFP-tubulin indicated that LtpM-labeled vesicles moved bidirectionally along microtubules (Fig. S11*B*). To validate this, we used the microtubule-depolymerizing drug nocodazole, as a control. Only diffuse undirected vesicle movement with the mean velocity of 0.07 ± 0.01 μm/s (*n* = 7) was observed in nocodazole-treated cells (Movies S5 and S6). pmCherry–LtpM and -LtpM NxN were expressed at the same level in the transfected HeLa cells indicating that the inhibitory effect of LtpM resulted from the glucosyltransferase activity of the effector and not from the higher expression of the WT compared with the inactive mutant (Fig. S11*C*).

## Discussion

*L. pneumophila* produces hundreds of Dot/Icm T4SS effectors to ensure its survival and replication within the LCV in phagocytic host cells. Less than 10% of the identified effectors have been studied in detail. Here, we characterized the *L. pneumophila* effector LtpM, which, based on similarity to the effector LtpD, was predicted to be a new modular Dot/Icm T4SS effector (28). We verified that LtpM is translocated by the Dot/Icm T4SS into target cells and demonstrated that the C-terminal domain of LtpM (amino acids 469–639), which shares 64% sequence identity with LtpD (amino acids 472–679) (28), selectively binds PI3P with an affinity of 591 nm and directs LtpM to membranes. Moreover, co-expression studies revealed co-localization of LtpM with well-characterized biosensors for PI3P (p40^Phox^) and PI(3,5)P_2_ (MLIN1), whereas co-localization with biosensors for PI4P, PI(3,4)P_2_, and PI(3,4,5)P_3_ or PI(4,5)P_2_ was not observed.

The use of PIP-binding domains for membrane anchoring and subcellular targeting has been established as common strategy for an increasing number of *L. pneumophila* effector proteins (39). PI4P-binding domains of DrrA/SidM (40) and SidC (41) and the PI3P-binding domain of RavZ (24) were structurally and functionally characterized in detail. Notably, structural homology modeling did not match the lipid-binding domain of LtpM to any of these bacterial or other eukaryotic PIP-binding domains or showed striking sequence similarities to the PI3P-binding domains of the effectors LidA (21), LpnE (42), RidL (43), and SetA (26). This indicates that the PI3P-binding domains of LtpM and LtpD, which are also found in dozens of other proteins across *Legionella* isolates, belong to a new family, which has been acquired or evolved independently from previously described domains.

In all previously characterized effectors, the PIP-binding domains are variably combined with one or more domains with diverse functions, for example proteases (24) or ubiquitin ligases (41), that manipulate host targets. The N-terminal domain of LtpM does not bear high homology to LtpD or conserved domains recognized by the PFAM database (44); however, we here discovered that it shares similarity to glycosyltransferase toxins, including the *L. pneumophila* effectors SetA (26) and Lgt1–3 (38, 45), *P. asymbiotica* toxin PaTox (33), and the family of large clostridial glucosylating toxins (*e.g. C. difficile* toxins TcdA and TcdB) (46).

We demonstrated that LtpM possesses glucohydrolase activity and prefers UDP-glucose as a substrate, which results in auto-glucosylation but also modification of artificial substrates such as BSA *in vitro*. Remarkably, we show that addition of PI3P increased the glucosyltransferase activity but not the glucohydrolase or the auto-glucosylation activity of LtpM by about 5-fold. A half-maximal effect of PI3P was observed at the same concentration range as the affinity of PI3P binding to LtpM. This suggests that the binding of PI3P to LtpM not only directs the localization of the effector in the cell but also opens or unmasks a binding site for (protein) substrates. Recently, PIP or membrane binding was also reported to control the activities of SidC (41), RavZ (24), and RalF (47), indicating that this is a widely adopted mechanism and that there is evolutionary pressure, which drives the fine-tuning of temporal and spatial control of effector activities. If the need to protect itself against damage by uncontrolled modification of bacterial proteins prior to effector translocation or limiting host cell damage and detection by host defense surveillance systems are the driving forces for this will be an intriguing question for future investigation.

Our ectopic expression studies showed that LtpM is toxic for yeast and to a lesser extent for mammalian cells, suggesting that LtpM is less active in mammalian cells or that it targets processes, which are essential in yeast but not mammalian cells. The *Legionella* glucosyltransferases Lgt1–3 are potent cytotoxins that inhibit protein synthesis (29, 38). We did not detect inhibition of protein synthesis by LtpM. Instead, we found that LtpM localizes to and reduces the mobility of Rab5- and Rab7-containing early and late endosomes on microtubules in a glucosyltransferase-dependent manner. As LtpM localizes to the LCV during infection, these data point to a role in the manipulation of the interaction of the LCV with the endosomal pathway and/or the movement of the LCV. Several effectors have already been implicated in the manipulation of these pathways, for example LegK2 and VipD, which interfere with the association and fusion of early endosomes with the LCV (48, 49); VipA, which derails organelle trafficking by modulating actin polymerization (50); and LegG1, which alters microtubule dynamics and LCV movement through the small GTPase Ran (51). Our observation that the chromosomal deletion of *ltpM* did not affect the proliferation of *L. pneumophila* in different model hosts phenocopies findings for numerous other Dot/Icm T4SS effector deletion mutants and is consistent with the hypothesis that LtpM might target host cell processes that are already heavily manipulated by additional effectors (13, 52).

Overall, LtpM resembles the *L. pneumophila* glycosyltransferase effector SetA in many aspects, including domain structure as well as localization and cytotoxicity upon ectopic expression in eukaryotic cells (26). Sequence alignment and structural homology modeling showed that LtpM shares several conserved amino acids with other glycosyltransferases; however, the absence of a conserved DxD motif, which is considered a hallmark of classical GT-A–type glycosyltransferases and so far is found in all bacterial glycosyltransferase toxins, including SetA, distinctly distinguishes LtpM.

The DxD motif is involved in Mn^2+^, UDP, and glucose binding and typically is of critical importance for activity. In many toxins, *e.g.* TcdB and PaTox, the second aspartate of the DxD motif directly coordinates the divalent cation, whereas the first coordinates via a water molecule and also interacts with the hydroxyl groups of UDP-ribose and with glucose to position UDP-glucose accurately in the catalytic cleft of the enzyme (33, 37, 53). This is supported by aromatic stacking between a tryptophan residue and the uracil ring of the activated sugar. Although Trp-13 of LtpM is placed to fulfill a similar function, a DxN instead of the classical DxD motif is present. This DxN motif is conserved in a large number of proteins containing LtpM-like domains across various *Legionella* species. Our experiments showed that despite this change, LtpM possesses glucohydrolase and glucosyltransferase activity and, remarkably, that this activity does not require mono- or divalent cations.

Few metal-independent GT-A–type glycosyltransferases, for example the sialyltransferases from family GT42 (54) and β-1,6-GlcNAc transferase C2GnT-L (55), have been reported. The metal-independent glycosyltransferase BoGT6 of the intestinal commensal *Bacteroides ovatus* possesses an NxN instead of a DxD motif (56, 57). It has been proposed that metal-independent glycosyltransferases use basic amino acids or the hydroxyl group of tyrosines to stabilize the substituted phosphate leaving group (37). In LtpM, exchange of lysine 123 to serine blocked the enzyme activity, indicating that this residue might fulfill a similar function. Notably, conversion of the DxN motif of LtpM into either a classical DxD or a BoGT6-like NxN motif both rendered LtpM inactive, demonstrating that the full DxN motif is essential for catalysis and suggesting that the active site is optimized for this motif and not a degenerated derivative of any previously described glycosyltransferase domains. Future mutational and structural studies of LtpM will expose its active-site geometry and catalytic mechanism.

Taken together, here we reveal that LtpM is the prototype of a new, widely distributed family of *Legionella* Dot/Icm T4SS glucosyltransferase effectors, which is localized and activated by PI3P and exhibits a unique catalytic site structure, that, unlike any other known glycosyltransferase toxin, functions independently of metal ion cofactors.

## Experimental procedures

### Bacterial and yeast culture

Table 1 lists all bacterial and yeast strains used in this study. *Escherichia coli* strains were grown using LB broth or agar, supplemented with 30 μg/ml chloramphenicol, 100 μg/ml ampicillin, or 50 μg/ml kanamycin if the selection of plasmids was required. *L. pneumophila* strain Paris (16) was cultured at 37 °C on buffered charcoal yeast extract agar or in *N*-(2-acetamido)-2-aminoethanesulfonic acid-buffered yeast extract (AYE) liquid medium (58, 59). If required, *Legionella* media were supplemented with 25 μg/ml kanamycin and/or 6 μg/ml chloramphenicol. All chemicals and reagents were acquired from Sigma unless indicated otherwise.

**Table 1:** **Bacterial and yeast strains used in the study**

Strain	Serogroup/ genotype	Refs.
*L. pneumophila*		
Paris	O1; clinical isolate	16
Paris Δ*dotA*	*dotA* gene disrupted	73
Paris Δ*ltpM*	*ltpM* (*lpp0356*) gene disrupted	This study

*E. coli*		
Top10		Invitrogen
BL21-CodonPlus (DE3)-RIL	*E. coli* B F^−^ *ompT hsdS*(r_B_^−^ m_B_^−^) *dcm*^+^ Tet^r^ *gal* λ(DE3) *endA* Hte [*argU ileY leuW* Cam^r^]	Agilent Technologies

*S. cerevisiae*		
MH272-3fα	*ura3; leu2; his3; trp1; ade2*	Stratagene (Agilent, Frankfurt, Germany)
BY4741	*MATa; his3*Δ*1; leu2*Δ*0; met15*Δ*0; ura3*Δ*0*	74

*S. cerevisiae* MH272-3fα or BY4741 was grown at 30 °C on rich medium containing glucose (YPD: 1% yeast extract, 2% peptone, 2% glucose) or on minimal medium with the appropriate supplements (SD: 0.67% yeast nitrogen base without amino acids (Difco), 2% glucose; SGal: 0.67% yeast nitrogen base without amino acids, 2% galactose). The lithium acetate protocol was used to transform yeast (60). To assess the effect of expression of different LtpM variants, serial dilutions of yeast were spotted on SD and SGal medium. Growth was analyzed after 3 days of incubation at 30 °C.

### Cell culture

All human cell lines were purchased from the American Type Culture Collection (ATCC) and maintained in a humidified atmosphere of 5% CO_2_ at minimal passages. A549 lung and HeLa cervical epithelial cells were cultured in Dulbecco's modified Eagle's medium (DMEM) supplemented with 10% fetal bovine serum, GlutaMAX^TM^ (Invitrogen), and nonessential amino acids. Raw264.7 murine macrophage-like cells and human monocyte-like THP-1 cells were grown in Roswell Park Memorial Institute (RPMI) 1640 medium supplemented with 10% fetal bovine serum and GlutaMAX^TM^ (Invitrogen). THP-1 cells were differentiated by addition of 50 ng/ml phorbol 12-myristate 13-acetate for 72 h.

*D. discoideum* strain AX2-214 was obtained from dictyBase and grown static in HL5 liquid medium at 21–23 °C.

### Molecular biology

Table 2 summarizes the plasmids and primers used and generated for this study. All enzymes for DNA manipulation were obtained from New England Biolabs. Chromosomal DNA of *L. pneumophila* strain Paris (genome accession number NC_006368.1) was extracted using the Qiagen DNeasy blood and tissue kit and used to amplify *ltpM* (*lpp0356/lpp_RS01740*, protein accession number WP_011212979.1) by PCR. Point mutations were introduced into pRK5Myc *ltpM* with the QuikChange II site-directed mutagenesis kit (Agilent Technologies) and subcloned in other vectors as required. The sequence identity of all generated constructs was validated by sequencing. *L. pneumophila* was transformed with the plasmids by electroporation (61).

**Table 2:**
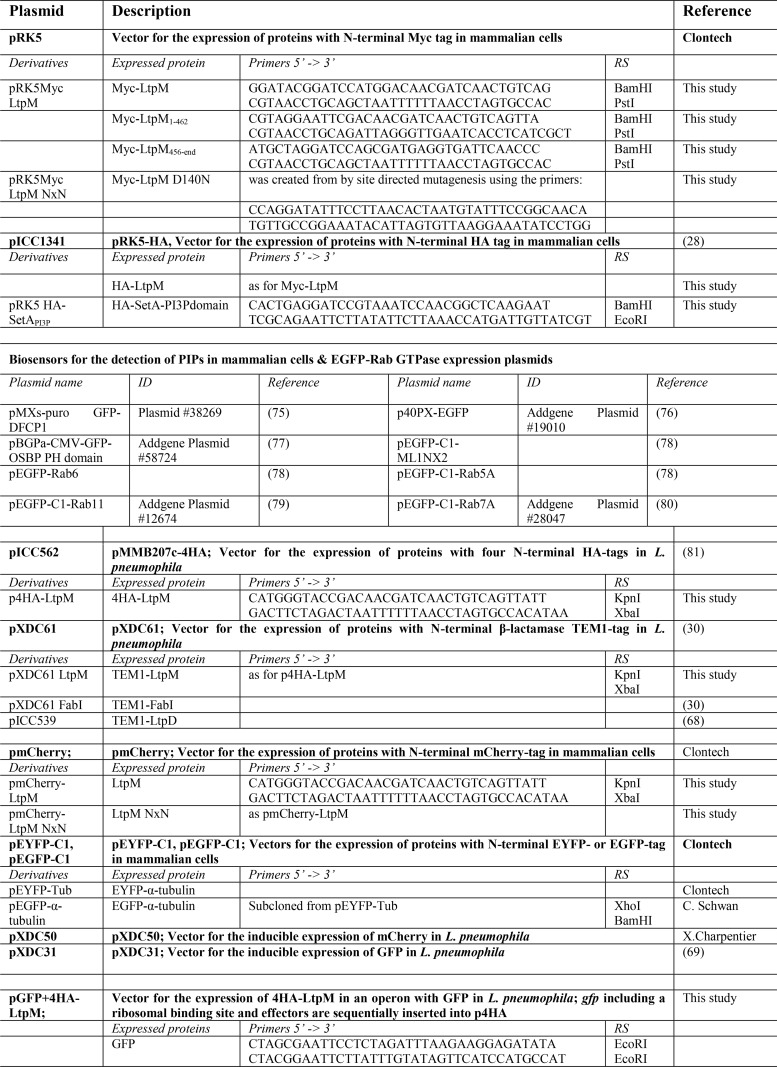

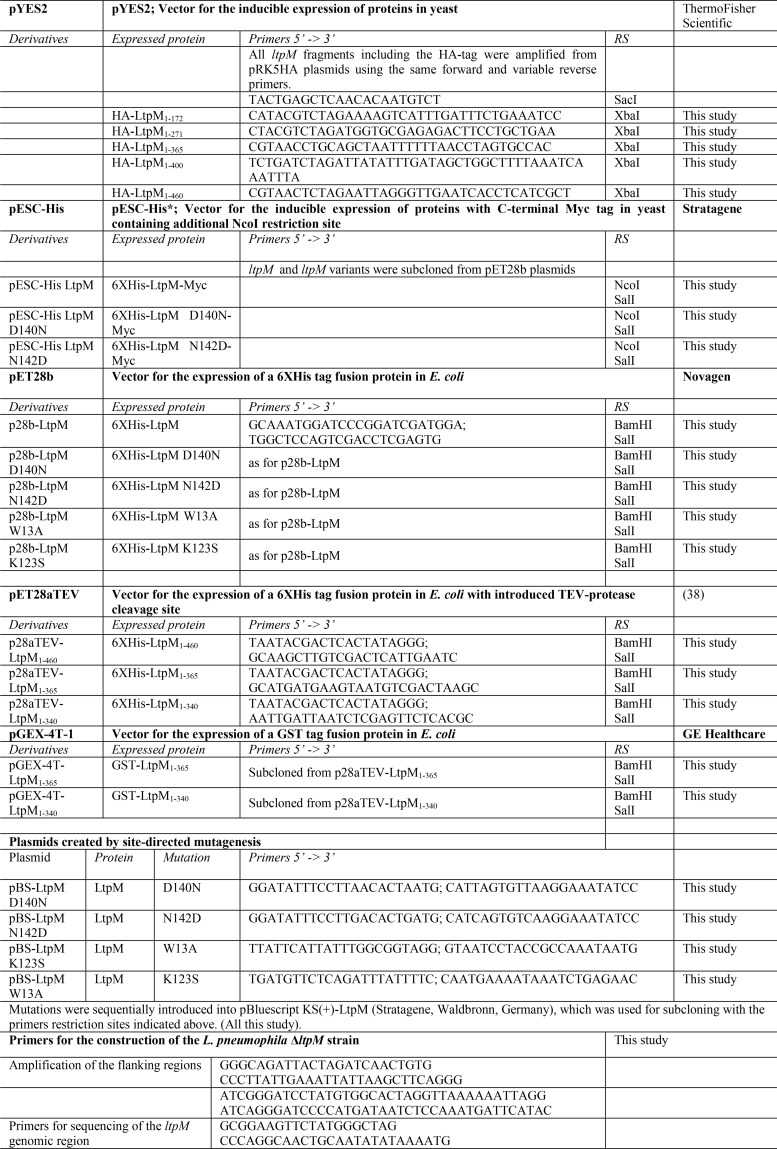
**Plasmids and primers used and created in this study** RS means restriction site. References 28, 30, 38, 68, 69, 75–81 are cited in the table.

To create the *L. pneumophila* Δ*ltpM* mutant, a kanamycin resistance (kan^R^) cassette was amplified from pSB315 (62) by PCR and inserted between bp 5′ and 3′ chromosomal flanking sequences of the *ltpM* gene. The entire construct was amplified by PCR and transformed into *L. pneumophila* by natural transformation (63). Deletion of the *ltpM* gene in kanamycin-resistant clones was confirmed by PCR and sequencing of the genomic region.

### Bioinformatics

The sequence of LtpM was analyzed for functional domains and motifs using SMART (64), the eukaryotic linear motif resource (65), and homologue proteins searched using NCBI Blast (https://blast.ncbi.nlm.nih.gov/Blast.cgi). Structural homology prediction was carried out using the Protein Homology/analogY Recognition Engine Version 2.0 (Phyre2 (66)). Protein accession numbers for sequence alignments were as follows: WP_010947098.1 (Lgt1/lpg1368); WP_010948548.1 (Lgt2/lpg2862); WP_061401042.1 (Lgt3/lpg1488); WP_105161403.1 (TcdA); WP_003418170.1 (TcdB); WP_010947694.1 (SetA/lpg1978); WP_015834366.1 (PaToxG); WP_058442075.1 (Lbru_2087); WP_058503838.1 (Lnau_0778); WP_028372515.1 (Llan_2410); WP_058495616.1 (Ldro_1318); WP_058501861.1 (Lisr_1509); WP_045094905.1 (Lfa_0737); and WP_058388204.1 (Lche_3126).

### Protein purification

*E. coli* (BL21 Codon Plus) bacteria were transformed with the desired plasmid and grown at 37 °C in LB broth supplemented with the corresponding antibiotics (50 μg/ml kanamycin and 50 μg/ml chloramphenicol) until an absorbance of *A*_600_ = 0.8. Protein expression was induced by 0.5 mm isopropyl β-d-thiogalactopyranoside (IPTG) (Roth, Karlsruhe, Germany), and bacteria were grown for an additional 5 h at 23 °C. The bacterial cells were harvested by centrifugation at 6000 rpm for 15 min and were lysed in a lysis buffer containing 10 mm Hepes (pH 7.4), 150 mm NaCl, 25 mm imidazole, 1 mm β-mercaptoethanol, 30 μg of DNase I, and protease inhibitor mixture cOmplete (Roche Applied Science, Mannheim, Germany). Bacterial lysate was extracted via high-pressure homogenization with an M-110P Microfluidizer® (Microfluidics, Westwood, MA). Cellular debris was removed via centrifugation (14,000 rpm for 1 h at 4 °C) and filtration with sterile Filtropur S 0.45 filters from Sarstedt (Nümbrecht, Germany). Recombinant His_6_-tagged LtpM protein (or mutants) was purified via fast protein liquid chromatography with an Äkta Purifier system and a nickel-nitrilotriacetic acid column. Buffer A (10 mm Hepes (pH 7.4), 150 mm NaCl, 1 mm β-mercaptoethanol) with an imidazole gradient reaching from 25 to 500 mm was used for protein elution. Removal of imidazole was achieved by using Sephadex G-25 columns (GE Healthcare) according to the manufacturer's instructions.

### Preparation of L. pneumophila for infection

*L. pneumophila* strains were prepared for infection as described previously (67). Briefly, 3-ml AYE cultures containing 6 μg/ml chloramphenicol were inoculated to an *A*_600_ of 0.2 and incubated at 37 °C shaking for 20 h before protein expression was induced by adding 1 mm IPTG, if required. 21 h post-inoculation, the early stationary phase bacteria were suspended in cell culture medium containing chloramphenicol (6 μg/ml) and added to cells at a multiplicity of infection (m.o.i.) of 0.5–100, as indicated for the specific assays below. Infections were synchronized by centrifugation at 600 × *g* for 5 min.

### Translocation assay

Delivery of LtpM through the Dot/Icm T4SS into host cells was measured using the β-lactamase (TEM1) reporter as described previously (68). *L. pneumophila* Paris carrying the pXDC61 LtpM expression or control plasmids were induced with 1 mm IPTG for 1 h and used to infect Raw264.7 cells at an m.o.i. of 40. 1 h post-infection, the infected cells were washed, and 20 μl of fresh CCF2-AM β-lactamase substrate (LiveBLAzer^TM^ FRET-B/G loading kit, Invitrogen) was added. 3 h post-infection, excess CCF2-AM was removed by washing; the fluorescence emission at 450 and 520 nm was recorded on a Fluostar Optima plate reader, and the 450:520 nm emission ratio as an indicator of protein translocation was calculated.

### Microplate growth assays

Intracellular growth of *L. pneumophila* in differentiated THP-1 cells was measured using a fluorescence microplate reader assay as described previously (67). 8.5 × 10^4^ THP-1 cells were seeded per well of a black clear-bottom 96-well plate and differentiated for 72 h. The medium was replaced with 100 μl of complete growth medium without phenol red, but containing 6 μg/ml chloramphenicol and 1 mm IPTG. Three wells per condition were infected with the indicated *L. pneumophila* strains carrying pXDC31 (GFP) or pXDC50 (mCherry) expression plasmids (69) at an m.o.i. of 0.1–0.5. 1 h post-infection, the cell supernatant was exchanged for medium containing 100 μg/ml gentamicin. After 1 h of incubation, the cells were washed three times with PBS and then kept in supplemented growth medium without phenol red. Fluorescence was measured every 2 h over a period of 72 h using a FLUOstar Optima plate reader (BMG Labtech) equipped with atmospheric control unit (37 °C, 5% CO_2_). To measure intracellular growth in *D. discoideum*, 8.5 × 10^4^ amoeba per well were seeded in Low Fluorescence Axenic Medium (LoFlo), and the assay was performed at room temperature as described for the THP-1 cells.

### Phospholipid overlay assay

Phospholipid overlay assays were performed with lipid strips and PIP arrays purchased from Echelon Biosciences (Salt Lake City, UT). Nitrocellulose membranes pre-spotted with different phospholipids were blocked with 3% fat-free BSA in 50 mm Tris-HCl (pH 7.4), 150 mm NaCl, 0.1% Tween 20 for 1 h at room temperature and incubated with 100 nm recombinant LtpM constructs in 2 ml of blocking buffer at 4 °C for 5 h. Binding of the proteins to lipids was visualized by immunodetection with primary anti-LtpM mouse polyclonal antibody and secondary anti-mouse IgG antibody (Table 3).

**Table 3:** **Antibodies used in this study**

Antibody (catalog no.)	Source	Supplier
EEA1 (ab50313)	Rabbit	Abcam (United Kingdom)
His-tag (2365)	Rabbit	New England Biolabs (Germany)
LtpM	Mouse	Y. Belyi, Gamaleya Research Center (Moscow, Russia)
Mouse IgG HRP-linked	Rabbit	New England Biolabs (Germany)
Rabbit IgG HRP-linked	Goat	Cell Signaling Technology
Rps9	Rabbit	Eurogentec (Belgium), gift of S. Rospert (Freiburg University)
α-Tubulin (DM1A)	Mouse	Santa Cruz Biotechnology (Germany)
*Legionella* (PA1-7227)	Rabbit	ThermoFisher Scientific (United Kingdom)
HA.11, clone 16B12 (MMs-101P-1000)	Mouse	Cambridge Bioscience (United Kingdom)
HA-tag (ab9110)	Rabbit	Abcam (United Kingdom)
Myc tag, clone 4A6 (05-724)	Mouse	Millipore (United Kingdom)
Alexa488-labeled/rhodamine RedX-labeled anti-rabbit or anti-mouse IgG	Donkey	Jackson ImmunoResearch (UK)

### Surface plasmon resonance spectroscopy

Interaction analysis of LtpM and PI3P was performed as described previously (26) at 25 °C using a Biacore X100 biosensor (GE Healthcare) equipped with an SA sensor chip (GE Healthcare). Shortly thereafter, 200 nm biotin-PI3P (Echelon Biosciences) in HBS-EP+ running buffer containing 10 mm Hepes (pH 7.5), 150 mm NaCl, 3 mm EDTA, and 0.05% Tween 20 was injected across individual flow cells at a flow rate of 10 ml/min until response units reached 106.9. Fresh ligand surfaces were equilibrated by injecting three 60-s pulses of 1.0 m NaCl and 25 mm NaOH. Buffer for the dilution of recombinant LtpM or BSA control was exchanged for the running buffer containing 10 mm Hepes (pH 7.5) and 160 mm KCl by gel filtration. 2-Fold serial dilutions of the analyte with concentrations ranging from 11 nm to 5.7 μm were applied to the chip for the contact time of 110 s with a flow rate of 50 μl/s with subsequent washing steps of 500 s. The sensor surfaces was regenerated with a single pulse of 2 m guanidinium chloride for 60 s. Binding responses were referenced by subtracting an average buffer response from at least two blank injections. Binding affinity was evaluated using the Biacore X100 evaluation software.

### UDP-sugar hydrolase assay

UDP-sugar hydrolysis was measured as described (70). Recombinant LtpM, LtpM mutants, SetA, or TcdB(1–546) were incubated with 10 μm UDP-[^14^C]glucose in a buffer containing 50 mm Hepes (pH 7.5), 3 mm MgCl_2_, 2 mm MnCl_2_, and 10 mm DTT in a total volume of 10 μl at 30 °C (if other conditions are not indicated). Buffer containing 50 mm Hepes (pH 7.5), 150 mm KCl, 3 mm MgCl_2_, 2 mm MnCl_2_, and 10 mm DTT was used for TcdB(1–546). Samples of 0.8 μl were taken at each time point and subjected to polyethyleneimine (PEI)-cellulose TLC (Merck) with 0.2 mm LiCl as mobile phase to separate products of UDP-glucose hydrolysis. The plates were dried and analyzed by autoradiography using Typhoon FLA 7000 phosphorimager (GE Healthcare). Quantification was performed in MultiGauge Version 3.0 software.

### Glycosyltransferase assay

2 μg of bovine serum albumin (BSA, New England Biolabs (Frankfurt, Germany)), as an artificial substrate, was incubated with recombinant LtpM or SetA and 10 μm UDP-[^14^C]glucose in a buffer containing 50 mm Hepes (pH 7.5), 3 mm MgCl_2_, 2 mm MnCl_2_, 10 mm DTT, and 1 μm PI3P diC8 (Echelon Biosciences) at 30 °C (if other conditions are not indicated). Total volume was 20 μl for the lysate glucosylation and 10 μl for the glucosylation of BSA. Labeled proteins were analyzed by SDS-PAGE followed by phosphorimaging (Typhoon FLA 7000, GE Healthcare, Freiburg, Germany).

### Transient transfection

GeneJuice (Novagen) or Lipofectamine 2000 (Invitrogen) reagents were used according to the manufacturer's guidelines to transfect HeLa cells with eukaryotic expression plasmids.

### Cell viability assay

HeLa cells seeded in 24-well plates were transiently transfected with 1 μg of a desired plasmid (pmCherry-C1-LtpM, pmCherry-C1-LtpM NxN, or empty pmCherry-C1). Transfected cells were incubated for 18 h at 37 °C prior to live-cell imaging. Time-lapse fluorescence and phase-contrast images were collected every 10 min during the incubation of the cells at 37 °C for 42 h in a Lionheart FX automated microscope (BioTek). One time-lapse movie per well was recorded. The images were analyzed for the number of attached transfected cells at the selected time points using Metamorph software.

### Immunofluorescence microscopy

Cells on coverslips were fixed with 3.2% paraformaldehyde (PFA), washed with D-PBS, incubated with 50 mm ammonium chloride, washed, and permeabilized with 0.1% Triton X-100. After blocking with PBS containing 2% (w/v) BSA and 2% (v/v) natural donkey serum, samples were stained sequentially with primary and secondary antibodies (Table 3) and mounted using ProLong antifade reagent (Invitrogen). DNA was visualized with Hoechst 33342 dye. Samples were analyzed using an Axio Z1 Imager microscope (Fig. 1). Co-transfected cells were imaged on a Zeiss Axiovert 200M inverted microscope (Carl Zeiss, Jena, Germany). Typically 50–100 cells were accessed per condition, and at least two independent biological repeats were carried out per experimental series. Representative images were deconvoluted and processed using AxioVision software.

### Cholera toxin B and transferrin trafficking assays

The transferrin recycling assay was carried out as described previously (71). Briefly, HeLa cells were transfected with either pmCherry, pmCherry–LtpM, or pmCherry–LtpM NxN expression plasmids. After 21 h, medium was replaced with serum-free DMEM containing 20 μg/ml transferrin-647 (Tfn-647, Invitrogen) and incubated at 37 °C for 1 h. Cells were washed with PBS to remove residual Tfn-647, and the medium was replaced with serum-free DMEM containing 100 μg/ml unlabeled holo-Tfn and incubated at 37 °C for 10, 30, or 60 min. At the indicated time points, cell were washed with ice-cold PBS, trypsinized, fixed with 3.2% PFA, and then analyzed by flow cytometry.

Cholera toxin B trafficking was analyzed in HeLa cells transfected for 48 h with plasmid pmCherry-C1-LtpM, pmCherry-C1-LtpM NxN, or pmCherry-C1 encoding for mCherry–LtpM, mCherry–LtpM NxN, or mCherry, respectively. Transfected cells were incubated with the minimal medium for 30 min at 37 °C before the treatment with fluorescently labeled subunit B of cholera toxin (CTxB–AlexaFluor488) (Biotium (Biotrend), Cologne, Germany). CTxB–AlexaFluor488 was diluted with fresh ice-cold minimal medium to a final concentration of 1 μg/ml. The cells were incubated with CTxB for 10 min on ice to allow binding to the cytoplasmic membrane. After that, the medium was exchanged for fresh minimal medium, and the cells were incubated at 37 °C for 0, 10, or 30 min. At the indicated time points, the cells were fixed with 4% PFA and subjected to fluorescence microscopy analysis. To quantify the trafficking of CTxB from the plasma membrane to intracellular compartments, at each time point the relative fluorescence intensity of CTxB per pixel, along a 150-pixel-long line intersecting the plasma membrane and entering about 100 pixels into the cell, was measured and plotted using Metamorph software.

### Vesicle speed measurement

HeLa cells plated on glass-bottom dishes were transiently transfected with the desired plasmids. 6 μg of pmCherry–LtpM or pmCherry–LtpM NxN plasmid and 3 μg of marker-carried plasmid were used for transfection. For live-cell imaging, cells were incubated in a homebuilt microincubator that provided a humidified atmosphere (6.5% CO_2_ and 9% O_2_) at 37 °C on a Zeiss Axiovert 200M inverted microscope (Carl Zeiss, Jena, Germany). Time-lapse images of moving vesicles were collected for 3 min with 1-s intervals with a digital camera (Coolsnap HQ, Roper Scientific, Tucson, AZ) driven by Visiview 4.0.0 (Visitron, Puchheim, Germany) imaging software. Images with the resolution of 15.5 pixels per μm were taken. Image analysis and generation of video clips were performed using the TrackMate plug-in (72) for ImageJ software. Vesicles on the images were detected using the algorithm based on Laplacian of Gaussian filtering with the estimated vesicle diameter of 0.7 μm and the intensity threshold adjusted manually for each image sequence.

## Author contributions

N. L., C. M., D. C., K.-N. T., K. A., and G. N. S. data curation; N. L., C. M., and D. C. formal analysis; N. L., C. M., D. C., K.-N. T., T. J., K. A., and G. N. S. validation; N. L., C. M., D. C., K.-N. T., and G. N. S. investigation; N. L. and G. N. S. visualization; N. L. and G. N. S. writing-original draft; N. L., C. M., D. C., T. J., G. F., K. A., and G. N. S. writing-review and editing; T. J., K. A., and G. N. S. conceptualization; T. J. resources; T. J., G. F., K. A., and G. N. S. supervision; T. J., G. F., K. A., and G. N. S. funding acquisition; T. J. methodology; T. J., K. A., and G. N. S. project administration.

## Supplementary Material

Supporting Information

## Footnotes

5 The abbreviations used are:

Dot/Icm: Defective in organelle trafficking/Intracellular multiplication
T4SS: type IV secretion system
PI3P: phosphoinositol 3-phosphate
GT: glycosyltransferase
PIP: phosphatidylinositol phosphate
PI(3,5)P_2_: phosphatidylinositol 3,5-bisphosphate
PI(3,4)P_2_: phosphatidylinositol 3,4-bisphosphate
PI(3,4,5)P_3_: phosphatidylinositol 3,4,5-trisphosphate
OSBP: oxysterol-binding protein 1
PH: pleckstrin homology
m.o.i.: multiplicity of infection
AYE: *N*-(2-acetamido)-2-aminoethanesulfonic acid-buffered yeast extract
IPTG: isopropyl β-d-thiogalactopyranoside
CTxB: cholera toxin subunit B
DMEM: Dulbecco's modified Eagle's medium
LCV: the *Legionella*-containing vacuole
IF: immunofluorescence
PEI: polyethyleneimine
PFA: paraformaldehyde
Tfn-647: transferrin-647.
