# Supplementary material for: The Legionella effector LtpM is a new type of phosphoinositide-activated glucosyltransferase
Source: J Biol Chem. 2018 Dec 20;294(8):2862–79. doi: 10.1074/jbc.RA118.005952 (PMC6393602; doi:10.1074/jbc.RA118.005952)
Supplement: Supporting Information [file supp_294_8_2862__index.html]

The Legionella effector LtpM is a new type of phosphoinositide-activated glucosyltransferase — L. pneumophila glycosyltransferase LtpM — The Legionella effector LtpM is a new type of phosphoinositide-activated glucosyltransferase — L. pneumophila glycosyltransferase LtpM — Supporting Information 

# The *Legionella* effector LtpM is a new type of phosphoinositide-activated glucosyltransferase

## Supporting Information

- Supporting Information (to be published online) - Revised supporting information
- Supporting Information (to be published online) - Excel-File Table S1
- Supporting Information (to be published online) - PDB-file of Phyre2 structure homology model of LtpM
- Supporting Information (to be published online) - Supplemental Movie M1
- Supporting Information (to be published online) - Supplemental Movie M2
- Supporting Information (to be published online) - Supplemental Movie M3
- Supporting Information (to be published online) - Supplemental Movie M4
- Supporting Information (to be published online) - Supplemental Movie M5
- Supporting Information (to be published online) - Supplemental Movie M6
