# Supplementary material for: The Legionella effector LtpM is a new type of phosphoinositide-activated glucosyltransferase
Source: J Biol Chem. 2018 Dec 20;294(8):2862–79. doi: 10.1074/jbc.RA118.005952 (PMC6393602; doi:10.1074/jbc.RA118.005952)
Supplement: Supporting Information [file supp_RA118.005952_140908_1_supp_254147_pjzqj3.pdf]

The *Legionella* effector LtpM is a new type of phosphoinositide-activated glucosyltransferase

Nadezhda Levanova, Corinna Mattheis, Danielle Carson, Ka-Ning To, Thomas Jank, Gad Frankel, Klaus Aktories, Gunnar N. Schroeder

Supporting information

1. Figure S1. Localization of translocated 4HA-LtpM in macrophage-like cells.
2. Figure S2. LtpM is dispensable for the replication of L. pneumophila in host cells.
3. Figure S3. LtpM binds PI3P.
4. Figure S4. $LtpM_{456\text{-}639}$ is sufficient to localize LtpM to PI3P-containing membranes.
5. Figure S5. AB. Phyre2 structure homology modelling of the LtpM glucosyltransferase domain & C. Coomassie-stained gel of purified LtpM fragments.
6. Figure S6. Effect of divalent ions on UDP-glucose hydrolase activity of LtpM.
7. Figure S7. Effect of divalent ions on the glucosyltransferase activity of LtpM.
8. Figure S8. $LtpM_{1\text{-}460}$ comprises the minimal glucosyltransferase domain required for inhibition if yeast growth.
9. Figure S9. LtpM has no effect on transcription/translation.
10. Figure S10. LtpM does not affect retrograde trafficking of Cholera Toxin B or transferrin-recycling pathways.
11. Figure S11. Analysis of the movement of LtpM-labeled vesicles in HeLa cells.

12. Table S1. LtpM-homologues identified by Blast (Excel-file)

13. File S1. PDB-file for Phyre2 structure homology model of the LtpM glucosyltransferase domain (Figure S5).

14. Supporting Movies: Time-lapse microscopy movies.
    Analysis of the movement of LtpM-labeled vesicles in HeLa cells.
    M1. Time-lapse movie (2 min, 10 frames per second) of a HeLa cell transfected with pmCherry-LtpM WT. Scale bar 10 µm.
    M2. Automatic recognition of vesicles and track building performed with the TrackMate plug-in (ImageJ Software) in a HeLa cell transfected with pmCherry-LtpM WT. Spotted vesicles are shown in magenta. Tracks are shown in yellow. Scale bar 10 µm.
    M3. Time-lapse movie (2 min, 10 frames per second) of a HeLa cell transfected with pmCherry-LtpM NxN. Scale bar 10 µm.
    M4. Automatic recognition of vesicles and track building performed with the TrackMate plug-in (ImageJ Software) in a HeLa cell transfected with pmCherry-LtpM NxN. Spotted vesicles are shown in magenta. Tracks are shown in yellow. Scale bar 10 µm.
    M5. Time-lapse movie (2 min, 10 frames per second) of a HeLa cell transfected with pmCherry-LtpM NxN and treated with nocodazol (10 µM) for 2 h. Scale bar 10 µm.
    M6. Automatic recognition of vesicles and track building performed with the TrackMate plug-in (ImageJ Software) in a HeLa cell transfected with pmCherry-LtpM NxN and treated with nocodazol (10 µM) for 2 h. Spotted vesicles are shown in magenta. Tracks are shown in yellow. Scale bar 10 µm.

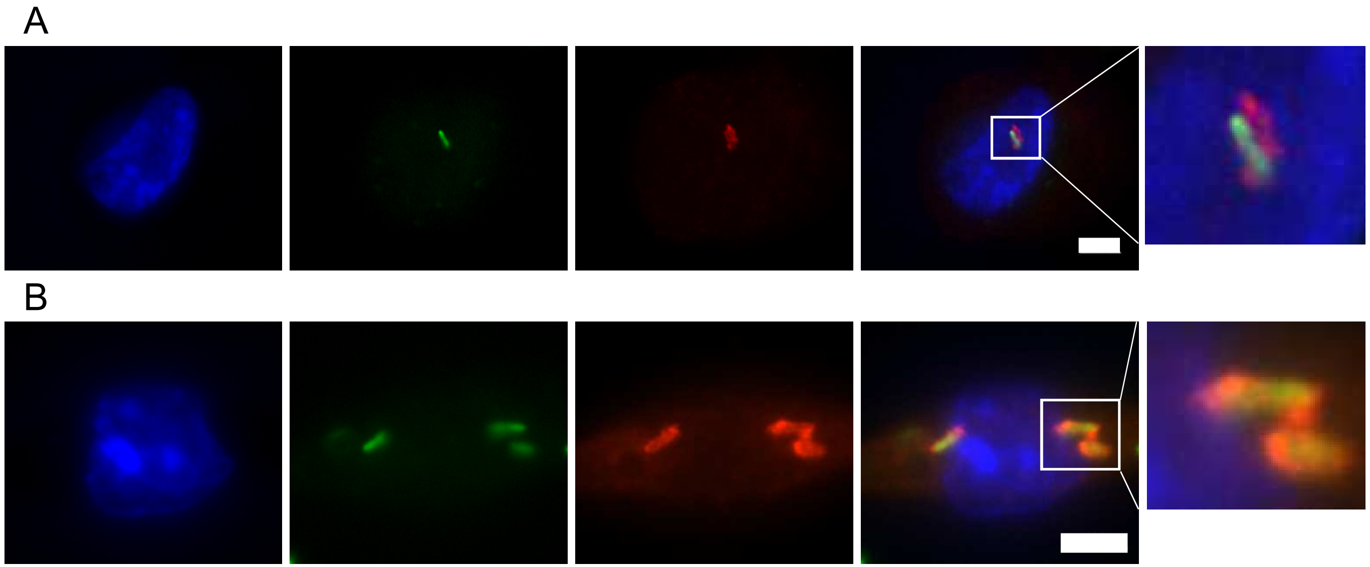

Figure S1. Localization of translocated 4HA-LtpM in macrophage-like cells. Differentiated human macrophage-like THP-1 (A) or murine Raw264.7 cells (B) were infected with *L. pneumophila* Paris expressing 4HA-LtpM and GFP. 6h post infection the cells were washed, fixed with PFA and processed for IF microscopy using anti-HA (red) antibody and DAPI to visualize DNA. Scale bar 5 µm.

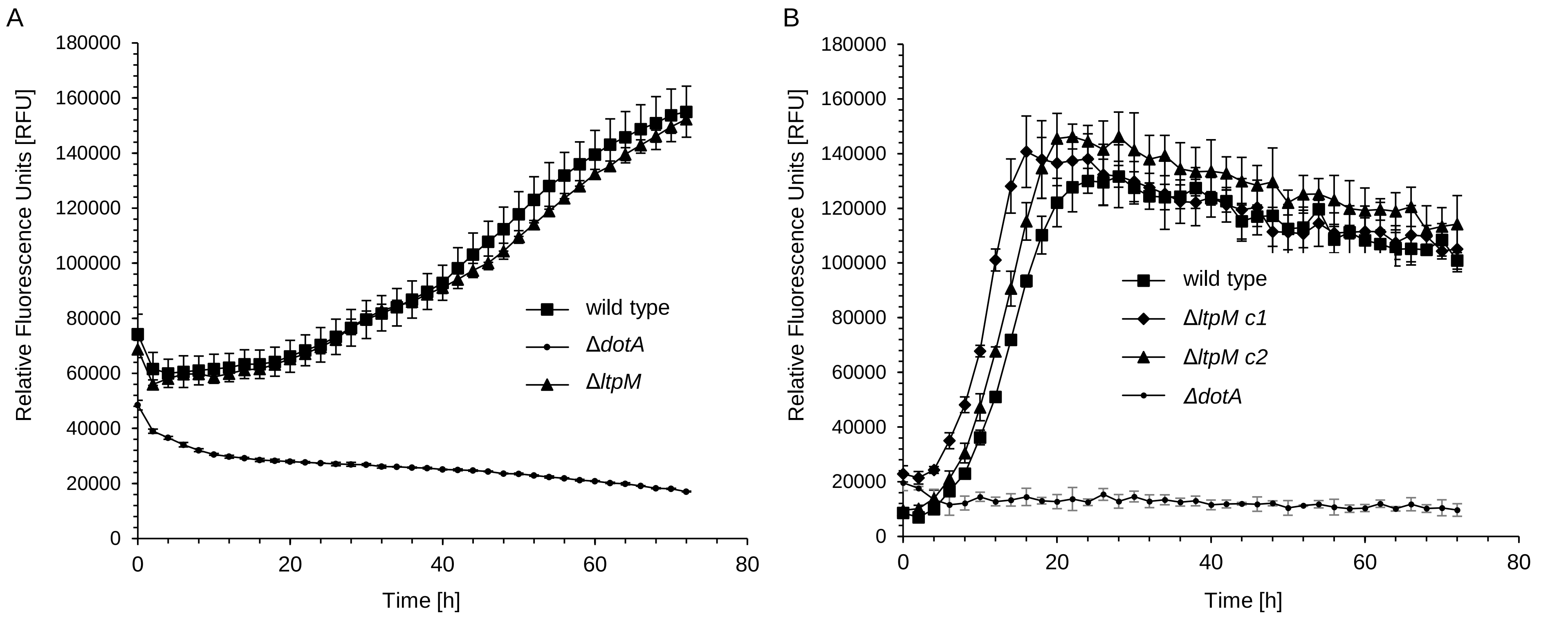

Figure S2: LtpM is dispensable for the replication of *L. pneumophila* in host cells.
Dictyostelium discoideum (A) or differentiated THP-1 macrophages (B) were infected with *L. pneumophila* Paris wild type, *ΔltpM* or *ΔdotA* expressing GFP (A) or mCherry (B) and fluorescence was measured every 2 h over a period of 72 h using a Fluostar Optima plate reader.

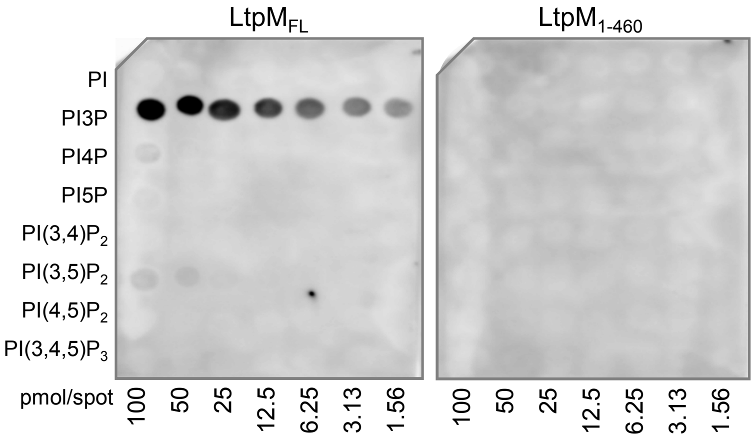

Figure S3. LtpM binds PI3P. PIP array with the full-length LtpM (LtpM$_{FL}$) and the catalytic domain LtpM$_{1-460}$. Nitrocellulose membranes pre-spotted with two-fold serial dilution of the indicated phosphatidylinositides (PIP) (100 to 1.56 pmol) were overlayed with 100 nM of the indicated protein. After extensive washing, proteins were detected with an anti-LtpM serum.

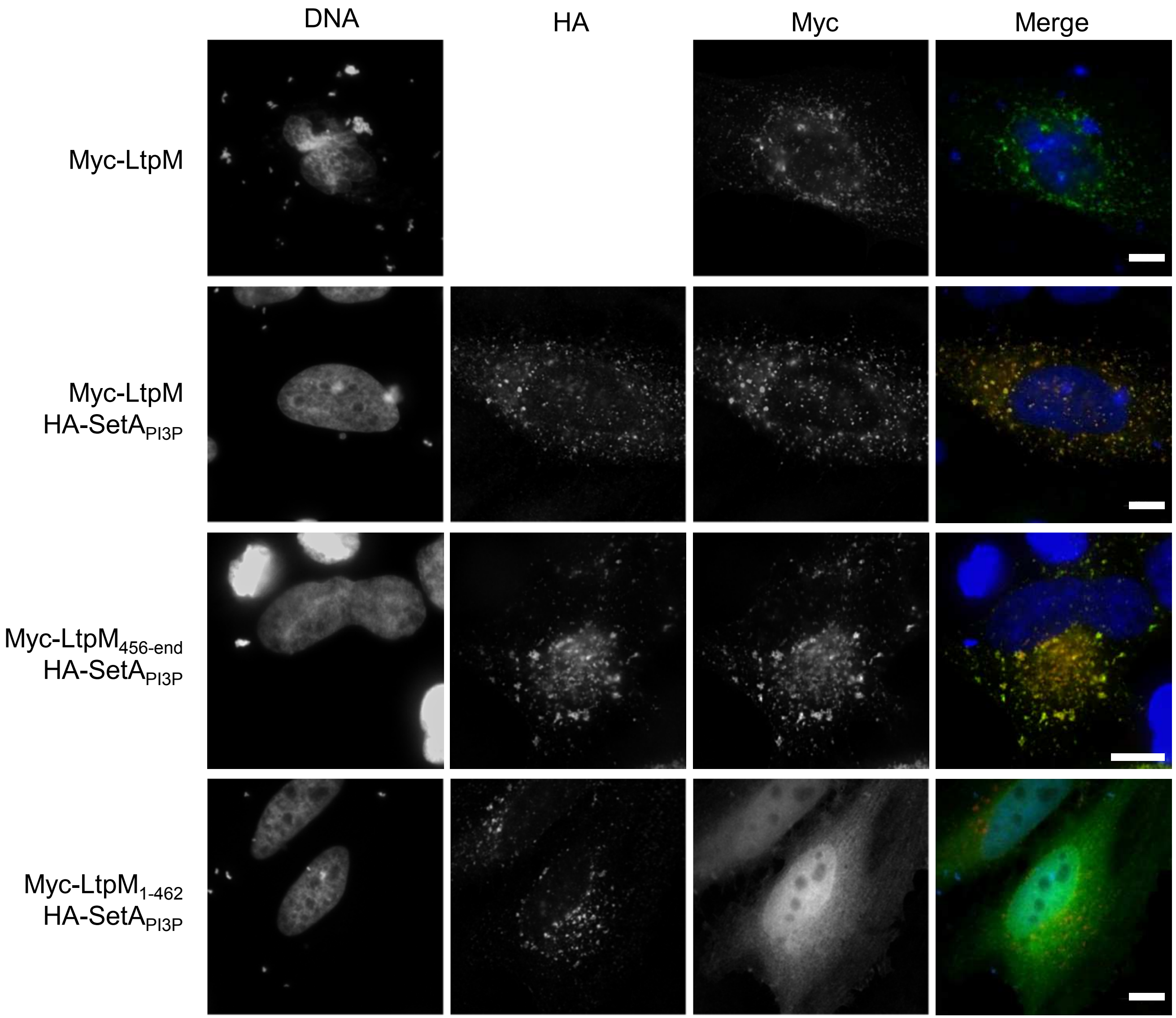

Figure S4: LtpM$_{456-639}$ is sufficient to localize LtpM to PI3P-containing membranes. HeLa cells were co-transfected with pRK5 plasmids encoding Myc-LtpM, -LtpM$_{1-462}$ or -LtpM$_{456-639}$ and HA tagged PI3P-binding domain of SetA (SetA$_{PI3P}$). 24h after transfection the cells were fixed and stained with anti-HA (red) and anti-Myc (green) antibodies and analyzed by IF microscopy. DNA was visualized with DAPI. LtpM and LtpM$_{456-639}$ localize to HA-SetA$_{PI3P}$ containing vesicular structures whereas LtpM$_{1-462}$ shows diffuse cytoplasmic distribution. Representative of at least 3 independent experiments. Scale bar 10 µm.

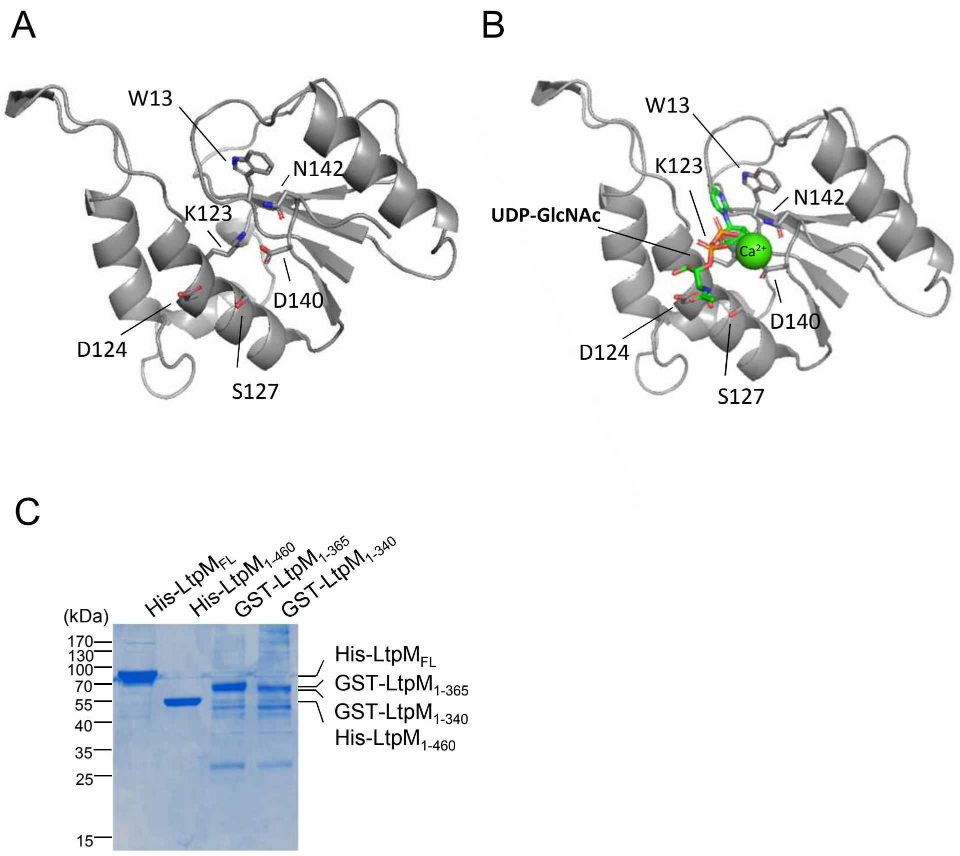

Figure S5. A, B. Phyre2 structure homology modelling of the LtpM glucosyltransferase domain. A. Phyre2 model of the fragment 9-145 of LtpM based on the crystal structure of PaTox-G from P. asymbiotica (PDB: 4mix). Model shows 59.9% confidence. DxN-motif (D140, N142) and conserved amino acids from the catalytic pocket (W13, K123, D124, S127) are shown in sticks. B. Model of coordination of UDP-GlcNAc and Ca^{2+} in the catalytic pocket of LtpM. **C. Purification of the LtpM fragments.** Coomassie-stained gel loaded with 3 µg of LtpMFL (His-tagged), $LtpM_{1-460}$ (His-tagged), $LtpM_{1-365}$ (GST-tagged) or $LtpM_{1-340}$ (GST-tagged).

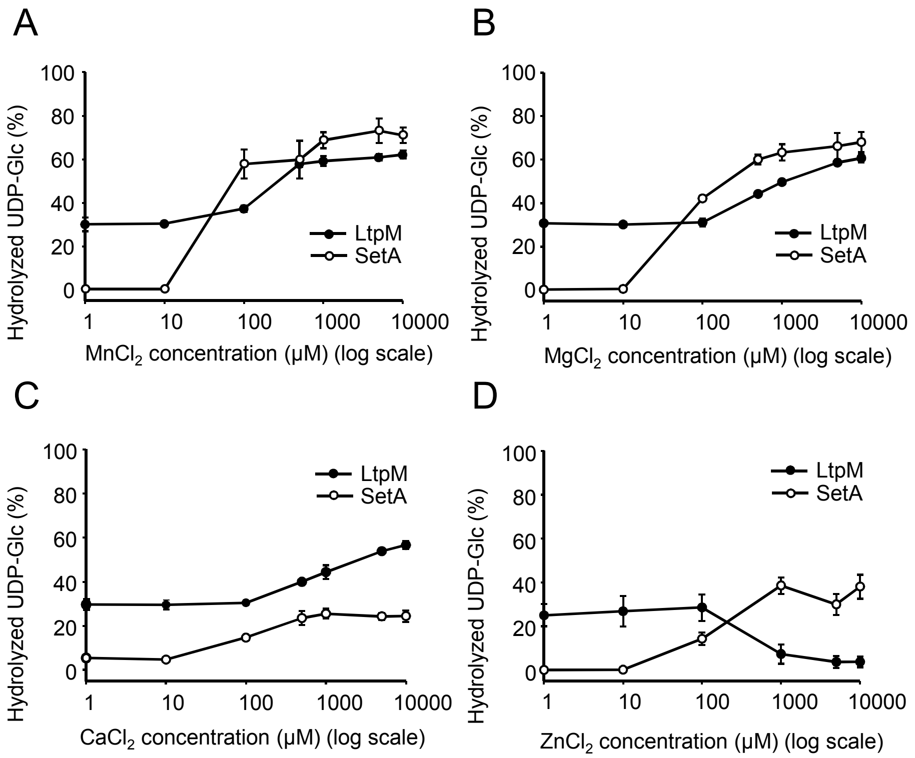

Figure S6. Effect of divalent ions on UDP-glucose hydrolase activity of LtpM. 1 µM LtpM (black circles) or SetA (white circles) were incubated with 10 µM UDP-[14C]Glc at 30°C for 30 min in a buffer containing 50 mM Hepes (pH 7.5), 10 mM DTT and 100 µM EDTA. Increasing concentrations of $MnCl_2$ (A), $MgCl_2$ (B), $CaCl_2$ (C) or $ZnCl_2$ (D) were added as indicated. Error bars indicate standard deviations of 3 independent experiments. The x axis represents logarithmic scale.

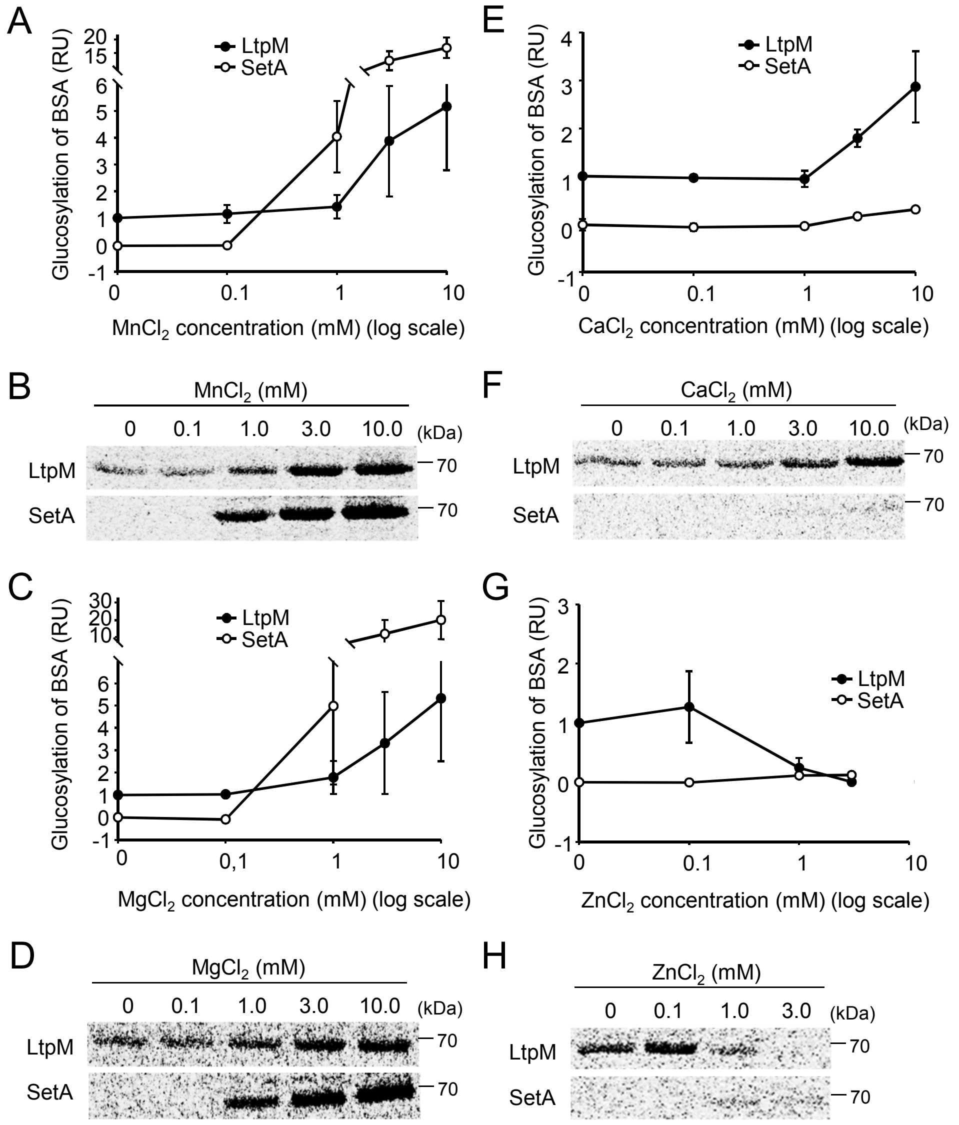

Figure S7. Effect of divalent ions on the glucosyltransferase activity of LtpM.
Glucosylation of BSA by LtpM (black circles) or SetA (white circles) (25 nM each) in the presence of different divalent ions. Reactions were performed at 30°C for 40 min in a buffer containing 2.5 µg BSA, 1 µM PI3P, 10 µM UDP-[14C]Glc, 50 mM Hepes (pH 7.5), 10 mM DTT and 1 mM EDTA. Increasing concentrations of $MnCl_2$ (A, B), $MgCl_2$ (C, D), $CaCl_2$ (E, F) or $ZnCl_2$ (G, H) were added as indicated. Representative autoradiograms are shown (B, D, F, H). Individual measurements were normalized to the value recorded for the reaction in the presence of LtpM without divalent ions added. Error bars indicate standard deviations of 3 independent experiments. The x axis represents logarithmic scale.

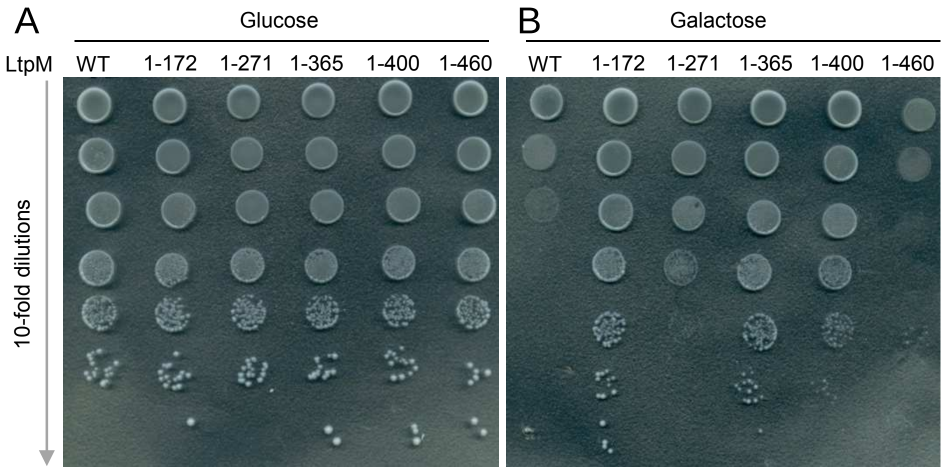

Figure S8. LtpM$_{1\text{-}460}$ comprises the minimal glucosyltransferase domain required for inhibition if yeast growth.
Serial 10-fold dilutions of *S. cerevisiae* BY4741 containing inducible pYes2 expression plasmids for LtpM or LtpM fragments were spotted on selective growth media containing either glucose (A) to repress or galactose (A) to induce expression of the LtpM variants. Images were taken after 3 days of growth at 30 °C. Representative of at least 3 independent experiments.

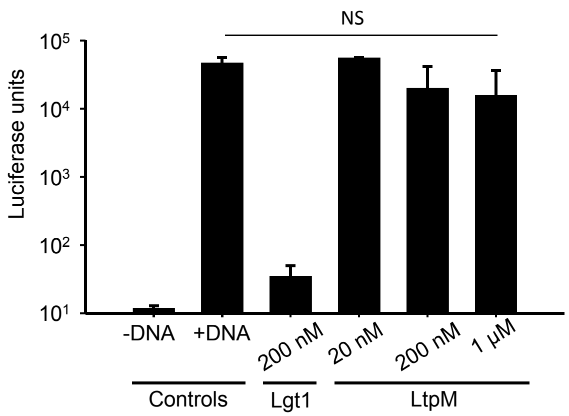

Figure S9. LtpM has no effect on transcription/translation.
Lgt1, but not LtpM, inhibits transcription/translation. Reactions were performed in the absence (controls) or presence of recombinant Lgt1 (200 nM) or LtpM (20 nM, 200 nM or 1 µM). Luciferase gene-encoding plasmid was used as matrix DNA. Reaction without matrix DNA was used as a negative control (-DNA). All measures were done in duplicate. Error bars indicate standard deviations of two biological replicates using two different purifications of His-tagged LtpM. The y axis represents logarithmic scale. NS, not significant.

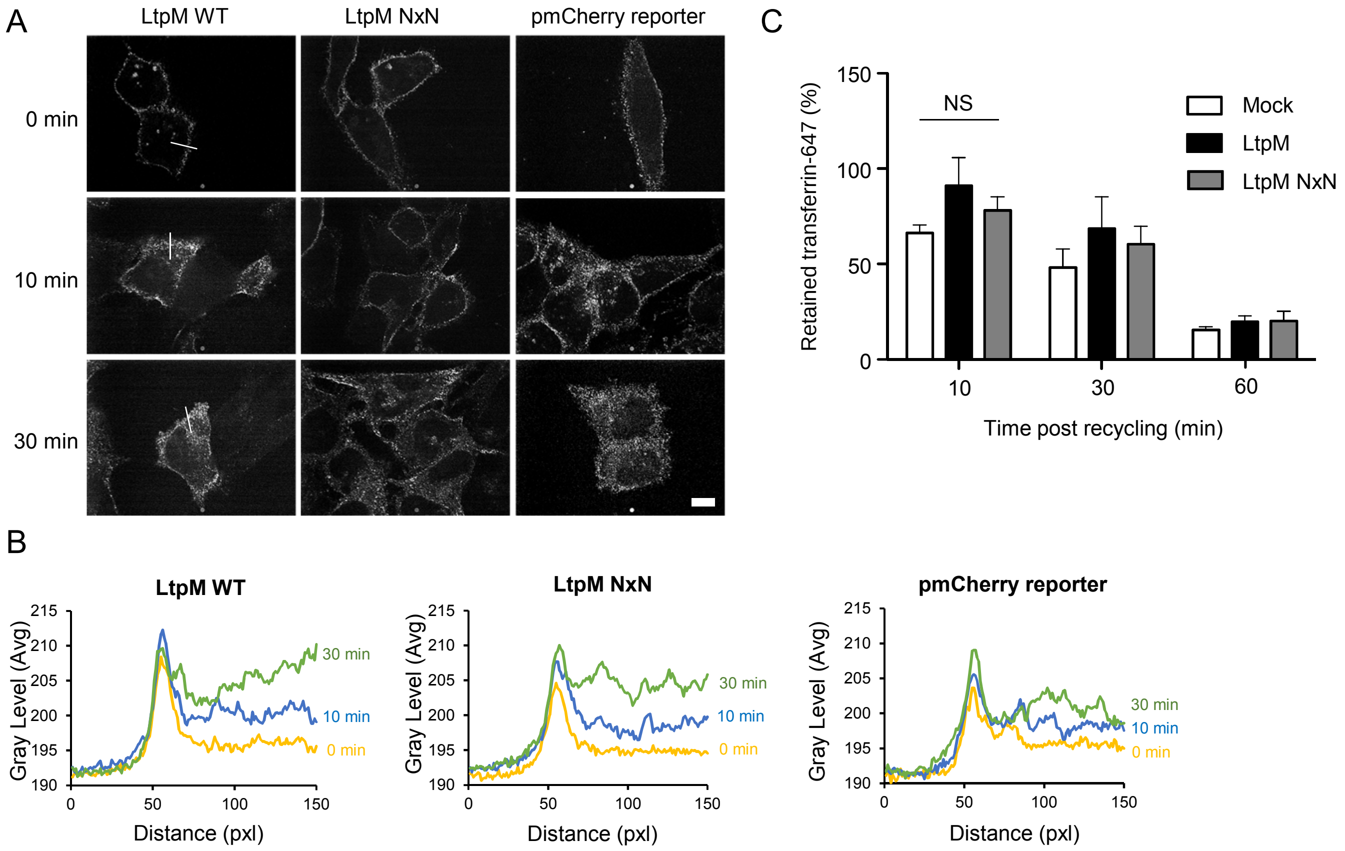

Figure S10. LtpM does not affect retrograde trafficking of Cholera Toxin B or transferrin-recycling pathways.

A. LtpM does not affect trafficking of CTxB from the cytoplasmic membrane to Golgi. Fluorescence images of HeLa cells transfected with pmCherry-C1-LtpM, pmCherry-C1-LtpM NxN or pmCherry-C1 (control) for 48 h followed by incubation with CTxB-AlexaFluor488 (grey scale) for 0, 10 or 30 min. Scale bar, 10 µm.

B. Overlays of the average relative fluorescence intensity (grey level) along a section crossing the cytoplasmic membrane (the peak at the position 50 pxl). A line represents overlays of at least 20 cells. Three independent experiments were performed.

C. LtpM does not affect recycling of transferrin (Tfn). HeLa cells expressing mCherry (Mock) or mCherry-fused to LtpM or -LtpM NxN were loaded with Tfn-647 and chased with unlabeled Tfn to promote recycling. At the indicated time points cells were fixed and cell-associated Tfn-647 fluorescence in mCherry-positive cells determined by flow cytometry. Average of the geometric mean for each sample taken and calculated as a % of T0 for each group. Statistical analysis including three independent experiments was conducted by two-way ANOVA with Bonferroni's multiple comparison test.

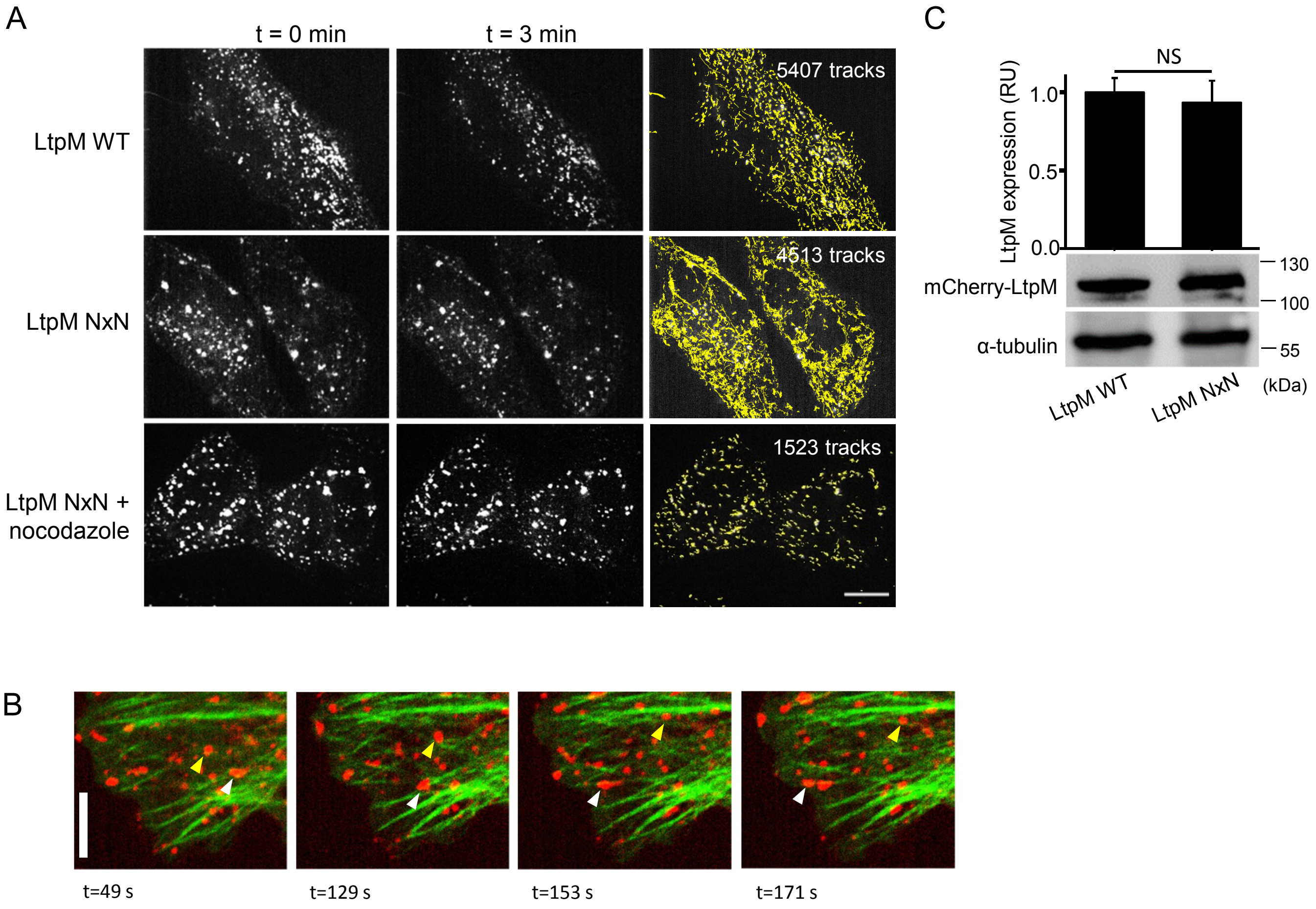

Figure S11. Analysis of the movement of LtpM-labeled vesicles in HeLa cells.

A. HeLa cells were transfected with LtpM WT or LtpM NxN (pmCherry) for 18 h. Nocodazol treated cells were used as a control. Nocodazol (10 µM) was applied for 2 h before microscopy. Single cell microscopy time-lapse movies (3 min, 1 frame per second) were recorded and the velocity of LtpM-labeled vesicles were analyzed (ImageJ Software). Track building is shown in yellow (right panel). The number of analyzed tracks in one time-lapse movie is indicated.

B. LtpM-labeled vesicles move bidirectionally along microtubules in HeLa cells. HeLa cells were transfected with LtpM-NxN (pmCherry, red) and α-tubulin (pEGFP, green) for 18 h. Single cell microscopy time-lapse movies (3 min, 1 frame per second) were recorded. Scale bar, 5 µm.

C. Western blot demonstrates production of pmCherry-LtpM and -LtpM NxN in HeLa cells. There is no significant difference in the production of the proteins. Antibodies against α-tubulin were used to control equal loading with the lysates. SD of 3 biological replicates are shown.
